# Spatio-Temporal Multiscale Analysis of Western Diet-Fed Mice Reveals a Translationally Relevant Sequence of Events during NAFLD Progression

**DOI:** 10.3390/cells10102516

**Published:** 2021-09-23

**Authors:** Ahmed Ghallab, Maiju Myllys, Adrian Friebel, Julia Duda, Karolina Edlund, Emina Halilbasic, Mihael Vucur, Zaynab Hobloss, Lisa Brackhagen, Brigitte Begher-Tibbe, Reham Hassan, Michael Burke, Erhan Genc, Lynn Johann Frohwein, Ute Hofmann, Christian H. Holland, Daniela González, Magdalena Keller, Abdel-latif Seddek, Tahany Abbas, Elsayed S. I. Mohammed, Andreas Teufel, Timo Itzel, Sarah Metzler, Rosemarie Marchan, Cristina Cadenas, Carsten Watzl, Michael A. Nitsche, Franziska Kappenberg, Tom Luedde, Thomas Longerich, Jörg Rahnenführer, Stefan Hoehme, Michael Trauner, Jan G. Hengstler

**Affiliations:** 1 Leibniz Research Centre for Working Environment and Human Factors, Department of Toxicology, Technical University Dortmund, Ardeystr. 67, 44139 Dortmund, Germany; myllys@ifado.de (M.M.); edlund@ifado.de (K.E.); hobloss@ifado.de (Z.H.); brackhagen@ifado.de (L.B.); begher-tibbe@ifado.de (B.B.-T.); hassan@ifado.de (R.H.); gonzalez@ifado.de (D.G.); zak@ifado.de (M.K.); marchan@ifado.de (R.M.); cadenas@ifado.de (C.C.); 2 Department of Forensic Medicine and Toxicology, Faculty of Veterinary Medicine, South Valley University, Qena 83523, Egypt; abdellatief-shakir@vet.svu.edu.eg; 3 Institute of Computer Science & Saxonian Incubator for Clinical Research (SIKT), University of Leipzig, Haertelstr. 16-18, 04107 Leipzig, Germany; friebel@izbi.uni-leipzig.de (A.F.); hoehme@uni-leipzig.de (S.H.); 4 Department of Statistics, TU Dortmund University, 44227 Dortmund, Germany; duda@statistik.tu-dortmund.de (J.D.); kappenberg@statistik.tu-dortmund.de (F.K.); rahnenfuehrer@statistik.tu-dortmund.de (J.R.); 5 Hans Popper Laboratory of Molecular Hepatology, Division of Gastroenterology and Hepatology, Department of Internal Medicine III, Medical University of Vienna, 1090 Vienna, Austria; emina.halilbasic@meduniwien.ac.at (E.H.); michael.trauner@meduniwien.ac.at (M.T.); 6 Department of Gastroenterology, Hepatology and Infectious Diseases, Medical Faculty at Heinrich-Heine-University, University Hospital Duesseldorf, 40225 Dusseldorf, Germany; mihael.vucur@med.uni-duesseldorf.de (M.V.); tom.luedde@med.uni-duesseldorf.de (T.L.); 7 MRI Unit, Leibniz Research Centre for Working Environment and Human Factors, Department of Psychology and Neurosciences, Technical University Dortmund, Ardeystr. 67, 44139 Dortmund, Germany; burke@ifado.de (M.B.); genc@ifado.de (E.G.); 8 Siemens Healthcare GmbH, 91052 Erlangen, Germany; lynn.frohwein@siemens-healthineers.com; 9 Dr. Margarete Fischer-Bosch Institute of Clinical Pharmacology, University of Tübingen, Auerbachstr. 112, 70376 Stuttgart, Germany; ute.hofmann@ikp-stuttgart.de; 10 Institute of Computational Biomedicine, Heidelberg University, Faculty of Medicine, Bioquant—Im Neuenheimer Feld 267, 69120 Heidelberg, Germany; christian.holland@bioquant.uni-heidelberg.de; 11 Histology Department, Faculty of Medicine, South Valley University, Qena 83523, Egypt; tahany_abbass@yahoo.com; 12 Department of Histology and Cytology, Faculty of Veterinary Medicine, South Valley University, Qena 83523, Egypt; esi.mohammed@vet.svu.edu.eg; 13 Department of Medicine I, University Hospital, 93053 Regensburg, Germany; andreas.teufel@medma.uni-heidelberg.de (A.T.); timo.itzel@medma.uni-heidelberg.de (T.I.); 14 Leibniz Research Centre for Working Environment and Human Factors, Department of Immunology, Technical University Dortmund, Ardeystr. 67, 44139 Dortmund, Germany; metzler@ifado.de (S.M.); watzl@ifado.de (C.W.); 15 Leibniz Research Centre for Working Environment and Human Factors, Department of Psychology and Neurosciences, Technical University Dortmund, Ardeystr. 67, 44139 Dortmund, Germany; nitsche@ifado.de; 16 Translational Gastrointestinal Pathology, Institute of Pathology, University Hospital Heidelberg, D-69120 Heidelberg, Germany; thomas.longerich@med.uni-heidelberg.de

**Keywords:** NASH, non-invasive imaging, transcriptomics, intravital imaging

## Abstract

Mouse models of non-alcoholic fatty liver disease (NAFLD) are required to define therapeutic targets, but detailed time-resolved studies to establish a sequence of events are lacking. Here, we fed male C57Bl/6N mice a Western or standard diet over 48 weeks. Multiscale time-resolved characterization was performed using RNA-seq, histopathology, immunohistochemistry, intravital imaging, and blood chemistry; the results were compared to human disease. Acetaminophen toxicity and ammonia metabolism were additionally analyzed as functional readouts. We identified a sequence of eight key events: formation of lipid droplets; inflammatory foci; lipogranulomas; zonal reorganization; cell death and replacement proliferation; ductular reaction; fibrogenesis; and hepatocellular cancer. Functional changes included resistance to acetaminophen and altered nitrogen metabolism. The transcriptomic landscape was characterized by two large clusters of monotonously increasing or decreasing genes, and a smaller number of ‘rest-and-jump genes’ that initially remained unaltered but became differentially expressed only at week 12 or later. Approximately 30% of the genes altered in human NAFLD are also altered in the present mouse model and an increasing overlap with genes altered in human HCC occurred at weeks 30–48. In conclusion, the observed sequence of events recapitulates many features of human disease and offers a basis for the identification of therapeutic targets.

## 1. Introduction

Non-alcoholic fatty liver disease (NAFLD), defined as >5% hepatocytes with fatty change, affects more than one billion people worldwide and represents a steadily rising cause of chronic liver disease [1,2,3,4]. An initially bland steatosis may progress to non-alcoholic steatohepatitis (NASH) and eventually to cirrhosis and hepatocellular carcinoma (HCC). Currently, no approved pharmacotherapies for NASH are available [5,6], and as a result, it represents the second most common indication for liver transplantation [7]. A big hurdle in the development of therapies is the lack of adequate mouse models that reliably recapitulate specific features of the human pathophysiology. These models are used in pre-clinical research to study the mechanisms of disease progression, identify therapeutic targets, and test therapeutic interventions [3,8,9]. Many different dietary mouse models have been established, and recently the ‘Western diet’ (WD) that mimics a fast-food-style diet has gained attention, because it mirrors several features of human NAFLD [10,11,12]. Key characteristics of the WD fed to mice include high fat (e.g., 40% kCal), high fructose (e.g., 20% kCal), high cholesterol (0.2–2%) [10,11], and the use of appropriate mouse strains such as C57BL/6 that are more susceptible to inflammation and fibrosis induced by obesogenic diets than, e.g., BALB/c or C_3_H/HeN mice [13]. 

A previously published landmark study reported that the WD mouse model recapitulates key physiological, metabolic, histologic, and transcriptomic changes observed in humans with NASH [14]. However, a time-resolved study to establish a sequence of critical events has not yet been performed, and pathophysiological changes relative to the duration of feeding needs to be further studied [15]. Moreover, a systematic comparison that identifies the similarities and differences to the human situation is not yet available. To bridge this gap, we performed a WD feeding study with 9 analysis time points over 48 weeks that encompasses the entire period from bland steatosis to advanced fibrosis and HCC, and have identified a sequence of the key events and analyzed their translational relevance. 

## 2. Materials and Methods

All authors had access to the study data and had reviewed and approved the final manuscript. Materials and resources used in this study are listed under Table 1.

### 2.1. Mice and Feeding Style 

Male 8-week-old C57BL6/N mice (Janvier Labs, France) were used. Immediately upon arrival, the mice were fed ad libitum with either ssniff R/M-H, 10 mm standard diet (SD), or with Western-style diet (WD) (Table 1) up to 48 weeks; ingredients of the used WD are listed under Table 2. The mice were housed individually under 12 h light/dark cycles at controlled ambient temperature of 25 °C with free access to water. Body weight changes were monitored and recorded once a week. An overview of the number of analyzed mice per experimental condition is given under Table 3. All experiments were approved by the local animal welfare committee (LANUV, North Rhine-Westphalia, Germany, application number: 81-02. 04. 2020. A304).

### 2.2. Induction of Acute-on-Chronic Liver Injury by Acetaminophen

Induction of acute liver injury by acetaminophen (APAP) was conducted as previously described [16,17]. Briefly, the mice were fasted overnight prior to intraperitoneal administration of a single dose of APAP (300 mg/kg b.w.), dissolved in warm phosphate-buffered saline (PBS). The mice were fed ad libitum after APAP injection. Analysis was undertaken in mice fed a WD for 48 till 53 weeks and no major difference was observed.

### 2.3. Induction of Acute-on-Chronic Liver Injury by LPS 

In order to induce apoptosis, 48-week WD-fed mice were challenged with lipopolysaccharides (LPS) from *E. coli* O55:B5 (4 mg/kg; intravenous) [18]. The livers were harvested 24 h after LPS injection and further processed for cleaved caspase-3 immunostaining.

### 2.4. Intravital Imaging

Intravital imaging of lipid droplets and macrophages in mouse livers was performed using an inverted two-photon microscope LSM MP7 (Zeiss, Germany), as previously described [19,20,21]. Briefly, after induction of anesthesia in mice [21], bolus tail vein injections of Hoechst 33258 (nucleus marker), TMRE/Rhodamine123 (markers of lobular zonation and mitochondrial membrane potential), Bodipy (lipid marker), and/or F4/80 antibody (macrophage marker) were performed approximately 30 min before the start of recording (Table 4). The time point 9 weeks was selected to represent the early stage (3–12 weeks) after WD feeding as the lipid droplet zonation was clearly visible. In order to investigate the functionality of ductular reaction, a bolus of the bile acid analogue CLF was administered intravenously via a catheter fixed in the tail vein. All recordings were performed in the left liver lobe. 

### 2.5. Magnetic Resonance Imaging (MRI) 

MRI was performed using a clinical 3Tesla MRI scanner with a clinical gradient system (80 mT/m, 200 T/m/s) using a dedicated receive coil (8 channel volumetric mouse array, Rapid Biomedical, Rimpar, Germany) with an inner diameter of 35 mm and a coil length of 80 mm. Anaesthetized mice were placed in the center of the coil and positioned in the isocenter of the magnet. Imaging parameters were based on routine clinical protocols to ensure clinical translation of findings and comparison with human clinical data but were optimized for spatial resolution.

#### 2.5.1. Tumor Detection

Dynamic contrast-enhanced imaging was performed by repeated acquisition of T1-weighted 3D gradient-echo images consecutively with a temporal resolution of approximately 10.5 s (coronal VIBE, TR 7.92 ms, TE 2.29 ms, flip angle 9°, 0.3 × 0.3 × 0.4 mm spatial resolution, accelerated using CAIPIRINHA with PAT factor 4). After the acquisition of three imaging volumes, a bolus (1 mL/kg b.w.) of the contrast agent gadoxetic acid was administered into the tail vein via a catheter, and acquisition of 3D T1-weighted GRE images continued for one hour. Signal intensity time courses were extracted from regions of interest (ROIs) placed in the liver, the gallbladder, the heart as well as the urinary bladder. 

#### 2.5.2. Estimation of the Fat Fraction

To estimate the fat fraction of the liver, coronal images were acquired using a 3D multi-echo gradient-echo Dixon pulse sequence (VIBE-q-Dixon, TR 10.6 ms, TE 1.54/2.97/4.40/5.83/7.26/8.69 ms, 0.5 × 0.5 × 1.2 mm spatial resolution, accelerated using CAIPIRINHA with PAT factor 4, 15 averages to compensate for respiratory motion). Fat fraction images were calculated using the automated postprocessing tools as implemented by the manufacturer (Software release VE11E) [22]. Fat fraction was analyzed in three ROIs in each liver of the WD- and SD-fed mice. 

#### 2.5.3. Assessment of Hepatocyte Uptake Capacity

T1-maps were acquired before, as well as after one hour of gadoxetic acid injection. Three-dimensional T1-weighted spoiled gradient echo images were acquired with different excitation flip angles (coronal VIBE, TR 7.92 ms, TE 2.44 ms, flip angle 2°/5°/15°/20°/25°, 0.3 × 0.3 × 0.4 mm spatial resolution, accelerated using CAIPIRINHA with PAT factor 4, 6 averages to compensate for respiratory motion). Pixel-wise nonlinear least-squares fitting was applied using home-built software in Python 3.8 and SciPy 1.7 to retrieve pre- and post-T1-maps. ΔT1-maps were calculated from pre- and post-T1-maps as ΔT1 is known to correlate with hepatocyte uptake and negatively correlates with liver function [23]. Relative change in T1 relaxation time was calculated (RE = ΔT1/T1pre, where T1pre reflects the T1-value before injection of contrast agent) to reflect liver function. Subtractions of pre- and post-contrast images acquired with flip angle 20° were used to visualize uptake of the contrast agent.

### 2.6. Sample Collection 

**Figure 1:**
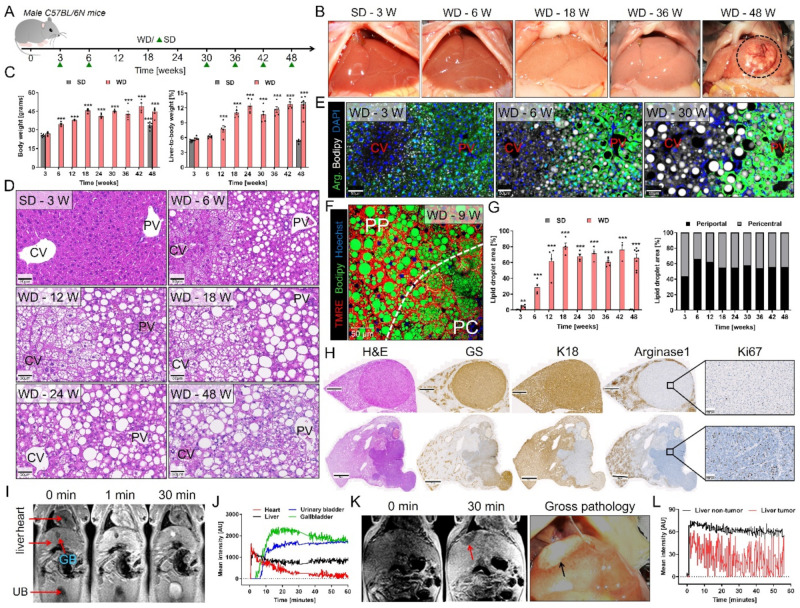
Lipid droplet accumulation and tumor development after Western diet feeding. (**A**) Experimental schedule indicating the number of weeks mice were on a SD or WD prior to analysis; green triangles: time periods with SD controls (details: Table 3). (**B**) Macroscopic appearance of the livers of mice on SD (week 3) and WD over 48 weeks. (**C**) Body weight and liver-to-body weight ratio. (**D**) Lipid droplet (LD) formation in H&E-stained liver tissue sections of mice fed a WD over 48 weeks; scale bars: 50 µm. (**E**) Zonation of LD formation. LD appear white, the periportal/midzonal regions are green due to immunostaining for arginase1 (Arg.); blue represents nuclear staining by DAPI; CV: central vein; PV: portal vein; scale bars: 50 µm. (**F**) Intravital visualization of LD using Bodipy (green). Differentiation of the periportal (PP) and pericentral (PC) lobular zones was achieved using the mitochondrial dye, TMRE, that leads to a stronger signal in the PP than the PC zone; scale bar: 50 µm (see also Videos S1 and S2). (**G**) Quantification of LD in relation to lobular zonation. Data in C and G represent the mean and standard error of 4–7 mice per time point. **: *p* < 0.01; ***: *p* < 0.001 compared to SD week 3, Dunnett’s (C) or Sidak’s (G) multiple comparisons tests; data of individual mice are illustrated by dots; SD: standard diet; WD: Western diet. (**H**) Immunostaining of a GS positive (upper panel; scale bars: 1 mm for whole slide scans and 100 µm for the closeup) and a GS negative (lower panel; scale bars: 2 mm for whole slide scans and 100 µm for the closeup tumor nodule from 48-week WD-fed mice for the hepatocyte marker K18, the periportal/midzonal marker arginase1, and the proliferation marker Ki67. (**I**) Stills from MRI analysis of a SD-fed mouse, week 48, before (0 min), as well as 1 and 30 min after injection of the contrast agent gadoxetic acid; GB: gallbladder. (**J**) Quantification of the gadoxetic acid-associated signal in the regions of interest indicated in I. (**K**) Visualization of hepatocellular carcinoma (HCC) that appears as a hypodense region (red arrow) in the liver of a WD-fed mouse at week 48 in comparison to the gross pathology of the same mouse. (**L**) Mean intensity of the MRI signal of gadoxetic acid in the tumor and non-tumor region of the liver after injection of gadoxetic acid.

Blood, as well as liver tissue samples, were collected time-dependently (Figure 1A) from defined anatomical positions of anesthetized mice, as previously described [24,25]. 

### 2.7. Liver Enzyme Assay 

Activities of transaminases (ALT and AST), as well as alkaline phosphatase (AP) in heart blood, were measured using the Piccolo Xpress Clinical Chemistry Analyzer (Hitado, Germany).

### 2.8. Histopathology, Immunohistochemistry, and TUNEL Staining

Hematoxylin and eosin (H&E), Sirius red, immunohistochemistry, as well as TUNEL stainings were performed in 4 µm thick PFA (4%)-fixed paraffin-embedded liver tissue sections using the Discovery Ultra Automated Slide Preparation System (Roche, Germany), as previously described [26,27]. Commercially available kits were used for staining of TUNEL (Promega, Germany) and Sirius red (Polysciences Europe GmbH, Germany), according to the manufacturers’ instructions. Immunohistochemistry was performed using the specific antibodies listed in Table 5. Following staining, whole slide scanning was undertaken using a digital scanner (Axio Scan.Z1, Zeiss, Germany). 

### 2.9. RNA-Seq Analysis 

Total RNA was extracted from frozen mouse liver tissue, using the RNeasy Mini Kit (Qiagen), according to the manufacturer’s instructions. DNase I digestion was performed on-column using the RNase-Free DNase Set (Qiagen) to ensure that there was no genomic DNA contamination. The RNA concentrations were determined on a Qubit™ 4 Fluorometer with the RNA BR Assay Kit (Thermo Fisher). The RNA integrity was assessed on a 2100 Bioanalyzer with the RNA 6000 Nano Kit (Agilent Technologies). All samples had an RNA integrity value (RIN) > 8, except three (6.9, 7.8, 7.9). Strand-specific libraries were generated from 500 ng of RNA using the TruSeq Stranded mRNA Kit with unique dual indexes (Illumina). The resulting libraries were quantified using the Qubit 1× dsDNA HS Assay Kit (Thermo Fisher) and the library sizes were checked on an Agilent 2100 Bioanalyzer with the DNA 1000 Kit (Agilent Technologies). The libraries were normalized, pooled, and diluted to between 1.05 and 1.2 pM for cluster generation, and then clustered and sequenced on an Illumina NextSeq 550 (2 × 75 bp) using the 500/550 High Output Kit v2.5 (Illumina).

### 2.10. Bioinformatics 

Transcript quantification and mapping of the FASTQ files were pre-processed using the software salmon, version 1.4.1, with option ‘partial alignment’ and the online provided decoy-aware index for the mouse genome [28]. To summarize the transcript reads on the gene level, the R package tximeta was used [29]. Differential gene expression analysis was calculated using the R package DESeq2 [30]. Here, a generalized linear model with just one factor was applied; this means that all combinations of diet (WD or SD) and time points (in weeks) were treated as levels of the experimental factor. The levels are denoted by SD3, SD6, SD30, SD36, SD42, SD48, WD3, WD6, WD12, WD18, WD24, WD30, WD36, WD42, and WD48. Differentially expressed genes (DEGs) were calculated by comparing two of these levels (combinations of diet and time point) using the function DESeq() and then applying a filter with thresholds abs(log_2_(FC)) ≥ log_2_(1.5) and FDR (false discovery rate)-adjusted *p* value ≤ 0.001. For pairwise comparisons, first, all time points for WD were compared against SD 3 weeks, which was used as a reference. Second, all time points for SD were compared against SD 3 weeks. Third, for all time points with data available for both SD and WD, the diet types were compared, e.g., WD30 vs. SD30. For the analysis of ‘rest-and-jump-genes’ (RJG, for a definition see below), the experiments were ordered in the (time) series TS = (SD3, WD3, WD6, WD12, WD18, WD24, WD30, WD36, WD42, WD48). Then, for every cutpoint in this series after WD3 and before WD36, two groups were formed by merging experiments before and after the cutpoint. Then, DEGs between the two groups were determined as described above, but for filtering abs(log_2_(FC)) ≥ log_2_(4) and an FDR-adjusted *p* value ≤ 0.05 was used. An additional filtering step was the use of an absolute correlation ≥ 0.9 between the expression profile of a gene and the corresponding RJG profile, e.g., (0, 0, 0,1, 1, 1, 1, 1, 1, 1) for a gene that ‘rests’ until week 6 and ‘jumps’ at week 12. K-means clustering was applied to cluster genes with respect to their expression profiles along the time series TS. Before applying k-means, a variance stabilizing transformation was applied and the top 1000 genes according to highest variance across all experiments in TS were preselected. Mean expression values across replicates were used as input for the clustering, with number of clusters set to k = 7. The number of clusters k = 7 was selected, since the values k = 3 and k = 7 yielded local optima, when the mean silhouette width, a cluster size validation measure, was plotted against k. Since k = 7 led to more accurately divided and biologically more plausible clusters, k = 7 was chosen. Gene set enrichment analysis (GSEA) was applied on the genes assigned to each cluster using the R package goseq, version 1.42 [31]. Overlaps of gene lists identified by differential expression analysis (DEGs) and gene lists associated with human liver diseases were calculated. Precision (number of genes in overlap divided by number of genes in human liver list) and recall (number of genes in overlap divided by number of DEGs in mouse data) were determined based on the databases of Itzel et al. [32] and on the database HCCDB by Lian et al. [33].

### 2.11. Western Blot Analysis and Quantification

Frozen liver tissue was homogenized in NP-40 lysis buffer using a tissue grind pestle to obtain protein lysates. These were separated by SDS-polyacrylamide gel electrophoresis (PAGE), transferred onto PVDF membranes, and analyzed by immunoblotting as previously described [34]. Membranes were probed with the following antibodies: MLKL, cleaved-Caspase-3, and GAPDH. HRP-conjugated secondary antibodies (anti-rabbit IgG and anti-mouse IgG) (Amersham) were used (Table 1). Intensity of the bands were analyzed using ImageJ software V1.8.0.

### 2.12. Quantification of Plasma Metabolites 

Amino acids, urea, pyruvate, fumarate, α-ketoglutarate, malate, and citrate were determined by GC–MS analysis as described previously [35,36]. The analysis was performed using 2.5 µL of mouse plasma collected from the portal and hepatic veins, as well as from the right heart chamber. Concentrations of ammonia were analyzed in whole blood samples immediately after collection from the portal and hepatic veins, as well as from the right heart chamber, using the PocketChem BA PA-4140 (Arkray, Inc., Edina, MN, USA) ammonia meter. 

### 2.13. Image Analysis 

The brightfield scans were segmented using the specialized whole slide image analysis software QuPath [37]. We interactively trained pixel-level classifiers at appropriate pixel resolutions (7.07, 3.54, or 0.44 µm) to segment all entities of interest such as tissue, Cyp2e1 or Sirius red positive regions. Structures within a 20 μm margin around tissue boundaries were excluded from analysis due to occasional staining and cutting artifacts. 

For segmentation of lipid droplets, we trained pixel-level classifiers using ilastik’s [38] pixel classification workflow. Due to frequent spatial aggregation of differently sized lipid droplets, a marker-based watershed transform was used for separation. The marker seeds were initialized with local maxima of pixel-precise Euclidean distances to the background. The resulting isolated lipid droplets were filtered based on size (>2.3 µm diameter) and roundness. Lipogranulomas (‘macrophage crowns’) were identified using ilastik’s object classification workflow. We used the post-processed lipid droplet and macrophage segmentations as input and trained an object classifier to separate ‘crowned’ and ‘naked’ lipid droplets, based on the amount of surrounding segmented macrophages. Lipogranulomas smaller than 4.42 µm diameter were excluded from analysis.

### 2.14. Patients 

A set of formalin-fixed paraffin-embedded liver tissue biopsies from 39 adult patients with NAFLD were acquired from the Medical University of Vienna. The biopsies were divided into four groups according to the fibrosis stage: fibrosis stage 0 (F0; *n* = 7), fibrosis stage 1 (F1; *n* = 10), fibrosis stage 2 (F2; *n* = 7), fibrosis stage 3 (F3; *n* = 9), fibrosis stage 4 (F4; *n* = 6). Patient characteristics are given under Table S1. The study was conducted in accordance with the ethical guidelines of the 1975 Helsinki Declaration and was approved by the local ethics committee.

### 2.15. Statistical Analysis 

Data were analyzed using Prism software (GraphPad Prism 9.1 Software, Inc., La Jolla, CA, USA). Statistical significance between experimental groups was analyzed using Dunnett’s/Tukey’s/Sidak’s multiple comparisons test, or unpaired *t* test, as indicated in the figure legends. 

## 3. Results

### 3.1. Spatio-Temporal Accumulation of Lipid Droplets and Tumor Development after Western-Style Diet Feeding

To study the time-course of pathophysiologically relevant events during Western diet (WD) feeding, we used an experimental schedule where 8-week-old male C57BL/6N mice were fed a WD or standard (SD) diet ad libitum for up to 48 weeks. Disease progression was analyzed in a time-dependent manner every 3–6 weeks (Figure 1A). Macroscopically, the livers of the WD-fed mice appeared pale as early as week 6, and some developed tumors after week 30 (Figure 1B). The WD-fed mice also rapidly gained weight until week 18, after which the weight plateaued (Figure 1C). The body weight of the SD-fed mice also increased over time up to week 30 and plateaued thereafter (Figure 1C; Figure S1); however, their liver-to-bodyweight ratio remained unaltered up to week 48 (Figure S1). In contrast, the liver-to-bodyweight ratio increased more than twofold in the WD-fed mice (Figure 1C). H&E-stained tissue sections showed the formation of lipid droplets (LD) in the WD-fed mice as early as 3 weeks after initiation, which became clearly visible after 6 weeks (Figure 1D,E). No LD were observed in livers of the SD-fed mice up to week 48 (Figure S2). Whole slide scans suggested a zonal pattern of LD at the early stage (weeks 3–6) after WD feeding (Figure S3A). To study the spatial distribution of LD, we stained the periportal/midzonal marker arginase1 and used the lipid dye Bodipy (Figure 1E). This analysis showed that large LD initially (week 3) developed in the midzonal compartment of the liver lobule, then progressed towards the periportal (weeks 6–12), and eventually to the pericentral zone (week 24 and later) (Figure 1E). The zonation was confirmed by staining for the pericentral marker Cyp2e1 (Figure S3B). Since tissue fixation may alter the actual size of LD and some may be lost during the washing steps, we next visualized the spatial distribution of LD intravitally in intact mouse livers with two-photon microscopy using the vital lipid dye Bodipy and the zonation marker tetramethylrhodamine ethyl ester (TMRE). This approach allowed us to visualize LD with improved resolution and demonstrated that microvesicular fatty change is present in the pericentral zone at early time points, in contrast to the macrovesicular LD observed in the midzonal/periportal regions (Figure 1F; Video S1). After WD feeding for longer than 24 weeks, this LD zonation was lost, and large LD appeared throughout the entire lobule (Figure S4; Video S2). The fraction of the total area covered by LD was quantified using whole slide images, differentiating between the pericentral (Cyp2e1 positive) and the midzonal/periportal (Cyp2e1 negative) lobular zones (Figure 1G). The LD area increased between weeks 3 and 12 and reached a plateau thereafter. This increase was more pronounced in the Cyp2e1 negative region, particularly until week 12; however, this zonal difference decreased at later time points (Figure 1G).

Macroscopically visible tumors were first observed at week 30 and later in 59% (16/27) of mice on WD. Histological and immunohistochemical analyses revealed tumor nodules with different morphological patterns, ranging between 0.2 and 10 mm of size (Figure 1H). Most nodules were highly differentiated, consisting of eosinophilic tumor cells with mild fatty change, sometimes surrounded by a rim of basophilic tumor cells (Figure 1H; Figure S5A). These nodules were glutamine synthetase (GS) and cytokeratin-18 (K-18) positive but arginase1 negative. The neoplastic hepatocytes were slightly smaller in size and less proliferative in comparison to hepatocytes in the surrounding non-tumor tissue (Figure 1H, upper panel; Figure S5A). Other tumor nodules were less differentiated and irregularly shaped. They consisted of small basophilic tumor cells without demonstrable fat inclusions and stained negative for GS, K18, and arginase1. Compared to the surrounding non-neoplastic hepatocytes, the proliferation was markedly increased (Figure 1H, lower panel; Figure S5B).

Since not all WD-fed mice developed tumors, we used MRI to non-invasively identify the tumor-developing mice. For this purpose, a bolus of the hepatobiliary-specific contrast medium gadoxetic acid (Primovist) was administered into the tail veins of 48-week WD- or SD-fed mice via a catheter, followed by dynamic imaging for 1 h. Within seconds after injection into the SD-fed mice, the contrast agent appeared in the blood circulation, was taken up by hepatocytes within 1–2 min, and homogeneously enriched in the liver parenchyma reaching a peak intensity within 10 min (Figure 1I,J). Approximately 5 min after administration, gadoxetic acid began to enrich in the gallbladder and urinary bladder. Similarly, homogeneous enrichment of gadoxetic acid was also observed in the liver parenchyma after administration into 48-week WD-fed mice without tumors (Figure S6A,B) but tumor nodules appeared hypointense relative to the surrounding non-tumor tissue (Figure 1K,L; Figure S6C) similar as reported for patients with HCC [39]. Subsequent macroscopic and histopathological analysis of the tumor-bearing mouse livers validated that the gadoxetic acid negative focal liver lesions were indeed HCC (Figure 1K; Figure S6C).

Taken together, WD feeding led to LD accumulation in hepatocytes which initially occurred in the midzonal and periportal compartments, prior to spreading throughout the liver lobule. After long-term feeding on the WD, 59% of the mice developed tumors which could be diagnosed non-invasively by gadoxetic acid-enhanced MRI.

### 3.2. Time-Resolved Genome-Wide Expression Analysis

**Figure 2:**
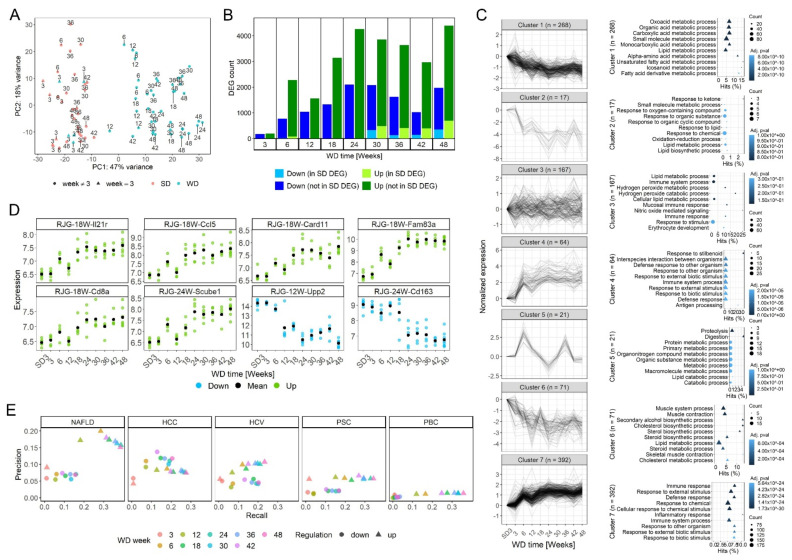
Time-resolved RNA_seq_ analysis. (**A**) Principal component (PC) analysis of all WD- (blue) and SD- (red) fed mice. Numbers in the panel indicate weeks on the WD or SD feeding. (**B**) Numbers of differentially expressed genes (DEGs) compared to SD week 3; adj *p* < 0.01; abs(log_2_ fold change) ≥ log_2_ (1.5). The light blue and light green color indicate DEGs that are differentially expressed in the WD and SD for the time periods with available SD controls (weeks 3, 6, 30, 36, 42, 48). (**C**) Left: k-means clustering of the 1000 genes with highest variability. In parentheses: numbers of genes making up the individual clusters. Right: 10 most enriched gene ontology (GO)-groups of each cluster. Count: number of DEGs in each GO-group and fdr-adjusted *p*-value; only GO-groups with at least three DEGs were included. (**D**) Examples of rest-and-jump genes (RJG). (**E**) Similarity of DEGs for the individual WD feeding periods compared to human NAFLD, hepatocellular carcinoma (HCC), hepatitis C virus infected liver tissue (HCV), primary sclerosing cholangitis (PSC), and primary biliary cholangitis (PBC) for up (∆) and down (•) regulated genes. (See also gene lists in Datasheet S1.)

To characterize the time-dependent changes of the transcriptomics landscape, liver tissues from the WD- and SD-fed mice (Figure 1A) were analyzed using RNA-seq. Principal component analysis (PCA) illustrated a large shift between WD weeks 3 and 6 along PC1, which explained 47% of the variance (Figure 2A). For longer periods, the sequential shift from one time point to the next along PC1 was smaller with a high degree of variability between individual mice. A shift along PC1 was also obtained for mice fed a SD, although the extent was much smaller. At all individually analyzed time points, the WD- and the age-matched SD-fed mice clearly clustered apart (Figure S7A). In a next step, we compared all time periods of WD and SD to the earliest time period of SD (SD week 3). Consistent with the PCA, the number of significantly up- or downregulated genes in the WD-fed mice strongly increased from week 3 to week 6, followed by only a moderate increase at the later time periods (Figure 2B; Datasheet S1). The color code indicates that only a small fraction of the genes that were differentially expressed in the WD-fed mice were also differentially expressed in the SD controls using SD week 3 as the reference. In contrast, most of the genes that were significantly deregulated in the SD-fed mice compared to SD week 3 were also significantly deregulated in the corresponding age-matched WD-fed mice (Figure S7B,C; Datasheet S1). 

To obtain an overview of the most frequent WD-induced gene expression changes over time, the 1000 genes that varied the most across all time points were analyzed by k-means clustering (Figure 2C). Downregulated genes appeared in clusters 1, 2, and 6, which mainly decreased at weeks 6 and 12, and were enriched in GO-groups associated with metabolic liver functions, including lipid metabolism. Clusters 4 and 7 contained upregulated genes that strongly increased at week 6, followed by a plateau, and were primarily enriched in genes associated with immune responses. An unusual time course was obtained for the genes summarized in cluster 5 that showed two peaks of increased gene expression at weeks 6 and 36, and contained GO-groups associated with proteolysis and protein metabolism. Finally, genes with relatively small expression changes were summarized in cluster 3. Since the k-means clusters mainly identified genes that increased or decreased already at week 6 and then remained altered compared to the control situation, we moreover aimed to specifically identify genes that remained unaltered in WD-fed mice (compared to SD week 3) until a specific time point—including also time points later than week 6—after which they became deregulated and remained so across subsequent time points, further called ‘rest-and-jump-genes’ (RJG). As expected, we observed numerous RJG at week 6 that were unaltered at week 3 and became deregulated starting at week 6 (Figure S7D). However, examples of RJG at weeks 12 and later illustrated that temporal expression patterns can be specifically identified also in cases where genes show lasting expression changes only after longer periods of WD feeding (Figure 2D; complete sets of RJG: Figure S7D). However, compared to the genes in the aforementioned k-means clusters, the numbers of RJG were relatively low, and were mostly associated with immune functions and T-cell subsets (Figure S7D). 

An important question to be addressed is the degree to which transcriptomic profiles in the WD mouse model resemble human NAFLD. To address this, using datasets of differential genes in human chronic liver diseases we calculated ‘recall’ as the fraction of differentially expressed genes in human NAFLD that are also deregulated in the present mouse model, and ‘precision’ as the fraction of genes deregulated in mice that are also differentially expressed in humans [26]. Compared to human NAFLD, a recall of 0.28 was obtained at week 6 for the upregulated genes in the WD mouse model that increased to 0.38 at week 48 (Figure 2E). In contrast, precision was 0.20 at week 6 but slightly decreased after longer periods on the WD (0.15 at week 48) (Figure 2E). Generally, precision and recall were much higher for the up- than for the downregulated genes. We hypothesized that the decrease in precision may be due to the progression to liver cancer, which could be reflected by a similarity to human HCC-associated genes. When compared to human HCC, recall increased from 0.18 (WD week 6) to 0.28 (WD week 48), while precision remained almost constant during this period of WD feeding. The downregulated genes resulted in a better recall and precision when compared to human HCC than to human NAFLD. Precision was lower in other chronic human liver diseases, such as primary sclerosing cholangitis (PSC) and primary biliary cholangitis (PBC) and appeared to be intermediate in hepatitis C virus infection (HCV). 

### 3.3. Progression from Simple Steatosis to Steatohepatitis

**Figure 3:**
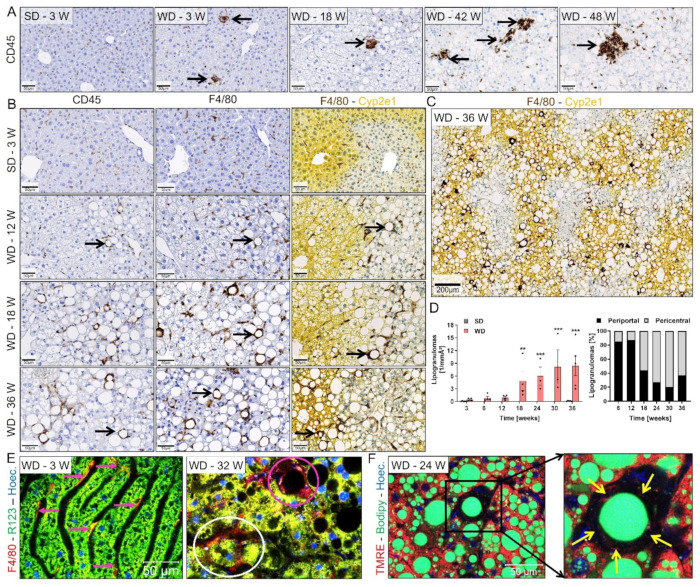
Formation of lipogranulomas (‘macrophage crowns’) after Western diet feeding. (**A**) Visualization of inflammatory foci by CD45 immunostaining. (**B**) Visualization of lipogranulomas (arrows) by immunostaining of CD45 and F4/80, and their lobular zonation by co-staining with the pericentral marker Cyp2e1; an overview image of WD week 36 is shown in (**C**). (**D**) Quantification of lipogranulomas’ density and zonation on whole slide scans in relation to the lobular zone; data represent the mean and standard error of 3–7 mice per time point. **: *p* < 0.01; ***: *p* < 0.001 compared to SD week 3. Unpaired *t* test; data of individual mice are illustrated by dots; SD: standard diet; WD: Western diet. (**E**) Intravital imaging of livers of WD-fed mice after intravenous injection of a fluorophore-coupled F4/80 antibody (red), the mitochondrial membrane potential marker Rhodamine123 (R123), and Hoechst for nuclear staining. The red arrows indicate Kupffer cells, the white circle shows a vital steatotic hepatocyte with mitochondrial and nuclear structures surrounded by F4/80 positive macrophages, and the pink circle indicates a lipid droplet enclosed by macrophages without discernible mitochondria or nuclear signal. (**F**) Intravital imaging of lipid droplets visualized by Bodipy; the yellow arrows indicate macrophages surrounding a lipid droplet. (See also Videos S3 and S4). Scale bars: 50 µm (**A**,**B**,**E**,**F**) and 200 µm (**C**).

Since progression from simple steatosis to NASH is characterized by a combination of lobular inflammation and hepatocellular ballooning leading to hepatocyte death [40], we next investigated if, and in which chronological order, these events occur in the present model. Well-known histological features of NASH are inflammatory foci which mostly consist of polymorphic granulocytes and some lymphocytes, and lipogranulomas (also called ‘macrophage crowns’) consisting of a fat vacuole surrounded by a layer of macrophages [41]. To study the kinetics of these features, inflammatory foci were visualized by CD45 and macrophages by CD45 and F4/80 immunostaining (Figure 3A–C) at different time intervals of WD feeding. A small number of inflammatory foci was already observed after 3 weeks (the shortest analyzed feeding period), remained relatively low until week 30, and strongly increased after week 36. Lipogranulomas were first observed at low levels at weeks 6 and increased at week 18 and later in WD-fed mice (Figure 3B–D), when the formation of LD had already reached a plateau (Figure 1G). Similar to the zonation of LD, the majority of lipogranulomas were initially localized to the midzonal/periportal lobular regions, and eventually shifted to the pericentral zone (Figure 3B–D). Lipogranulomas were further studied by intravital imaging after tail vein injections of antibodies against F4/80, the mitochondrial dye Rhodamine123, and the nuclei marker Hoechst. In line with the immunostaining data, only the resident Kupffer cells, and not lipogranulomas were detected in the livers of SD controls and early WD-fed (3 weeks) mice (Figure 3E; Video S3). However, F4/80 positive aggregates were clearly visible at week 12 and later in the livers of WD-fed mice (Figure 3E; Video S4A). Interestingly, two types of lipogranulomas were observed. Macrophages either encircled the remaining LD, as identified by Bodipy, while the cytoplasm and nucleus of the hepatocyte were no longer visible, as evidenced by the negative Rhodamine123/TMRE and Hoechst staining, respectively, or they enclosed viable steatotic hepatocytes (Figure 3E,F; Video S4B). This difference was difficult to detect in stained fixed tissues but was clearly visible in vivo after injection of the mitochondrial dyes, Rhodamine123 or TMRE, that discriminate live and dead cells.

**Figure 4:**
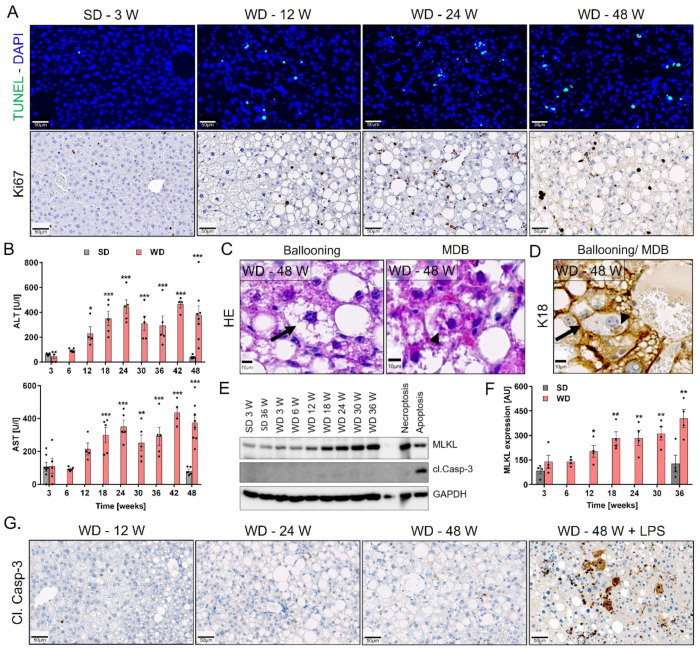
Cell death during NASH progression. (**A**) TUNEL and Ki67 staining in liver sections of SD- (3 week) and WD-fed mice. (**B**) Liver enzyme activities (ALT and AST) in the heart blood of mice fed a SD or WD. (**C**) Examples of ballooning (arrows) and Mallory–Denk bodies (arrowhead, MDB) in H&E-stained liver tissue sections. (**D**) Visualization of ballooning and MDB by K18 immunostaining. (**E**,**F**) Representative image of Western blot with accompanying quantification of the necroptosis marker MLKL and the apoptosis marker cleaved caspase-3 in livers of SD- and WD-fed mice over time. (**G**) Cleaved caspase3 immunostaining at different time intervals after WD feeding; LPS: lipopolysaccharide. Data in B and F are means and standard error of 4–5 mice per time point. *: *p* < 0.05; **: *p* < 0.01; ***: *p* < 0.001 compared to SD week 3, Dunnett’s multiple comparisons (**B**) or unpaired *t* (F) tests; data of individual mice are illustrated by dots; SD: standard diet; WD: Western diet. Scale bars: 50 µm (**A**,**G**) and 10 µm (**C**,**D**).

To investigate if the formation of lipogranulomas was associated with hepatocyte death, TUNEL staining was performed to detect DNA fragmentation, a common sign of different kinds of cell death. Positive TUNEL staining was evident starting at week 12 and later after WD feeding, thus paralleling the development of lipogranulomas (Figure 4A, upper panel). In agreement, transaminase activities were elevated in the blood of WD- but not SD-fed mice from week 12 onwards (Figure 4B). The observed increase in cell death was accompanied by replacement proliferation as evidenced by the positive staining for the proliferation marker Ki67 (Figure 4A, lower panel). In contrast to the early occurrence of cell death, full hepatocyte ballooning of K18 negative hepatocytes was not detected earlier than week 36 of WD feeding and was particularly obvious at week 48 (Figure 4C). This was evident by hepatocyte enlargement and rounding with rarefied cytoplasm in H&E staining (Figure 4C) and negative staining for the cytoskeleton marker K18 (Figure 4D). Hepatocyte ballooning was also accompanied by occurrence of K18 positive Mallory–Denk bodies (MDB) (Figure 4C,D). Next, we examined the responsible cell death form during the progression of NAFLD by analyzing the protein levels of the necroptosis marker, mixed lineage kinase domain-like (MLKL) protein, and the apoptosis marker, cleaved caspase-3, by Western blot. This analysis revealed a significant, time-dependent increase in MLKL protein levels in the liver of WD- but not SD-fed mice starting at week 12 and reaching a plateau after week 18 (Figure 4E,F). In contrast, protein levels of cleaved caspase-3 were not observed in both diets (Figure 4E). This was confirmed by immunostaining using antibodies against cleaved caspase-3, which showed negative staining even after 48 weeks of WD feeding (Figure 4G). As a positive control for apoptosis, immunostaining of cleaved caspase-3 was also performed in LPS-treated WD-fed mice, which showed a massive positive signal (Figure 4G).

Collectively, long-term feeding on WD led to the progression from simple steatosis to NASH, which was characterized by inflammatory foci, the formation of lipogranulomas, necroptotic hepatocyte death, replacement proliferation, and late during disease progression hepatocyte ballooning.

### 3.4. Ductular Reaction (DR) and Fibrosis Progression

**Figure 5:**
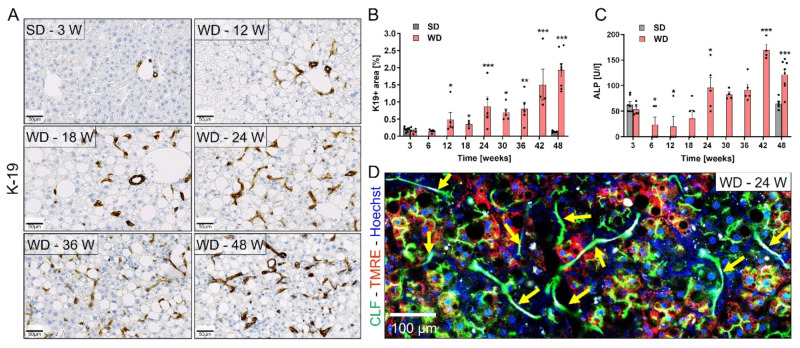
Development of bile-draining ductular reaction during NAFLD progression. (**A**) Immunostaining of the cholangiocyte marker K19 in liver sections of mice on SD (3 week) or WD over time. (**B**) Quantification of the K19 positive area. (**C**) ALP levels in blood of mice on SD or WD. (**D**) Intravital imaging after intravenous injection of the bile acid analogue CLF (green). Yellow arrows indicate ductular structures. Data in B and C represent mean and standard errors of 3–8 mice per time point. *: *p* < 0.05; **: *p* < 0.01; ***: *p* < 0.001 compared to SD week 3, Dunnett’s multiple comparisons test; data of individual mice are illustrated by dots; SD: standard diet; WD: Western diet; ALP: alkaline phosphatase; CLF: cholyl-lysyl-fluorescein. Scale bars 50 µm (**A**) and 100 µm (**D**).

In human NASH, continuous hepatocyte death triggers a DR [42]. To study if DR also occurred in the present model, K19 immunostaining was performed. In SD-fed mice, K19 staining was only observed in the bile ducts adjacent to the portal veins (Figure 5A; Figure S2). However, in WD-fed mice, a progressive DR was evident, starting at week 12 and increasing over time up to week 48 (Figure 5A,B). Development of DR was followed by elevated activities of alkaline phosphatase in the blood (Figure 5C). Whole slide scans demonstrated that the DR developed initially (weeks 12–18) in the periportal region, but later progressed towards the pericentral zone (Figure S8). Although they are believed to arise in order to replenish lost hepatocytes as part of a reparative process [43], the functional significance of such DR is still not clear. Thus, to investigate their function during NASH progression, we performed intravital imaging of the livers of WD-fed mice after tail vein injection of the green-fluorescent bile acid analogue CLF. Interestingly, CLF appeared in the lumens of bile canaliculi and DR within a few minutes after intravenous injection (Figure 5D). This observation would fit to a mechanism, where hepatocytes secrete CLF into bile canaliculi from where it reached the DR.

**Figure 6:**
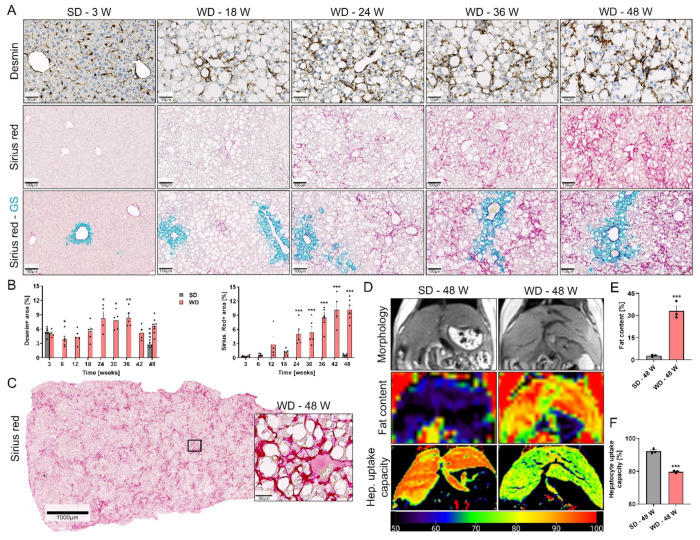
Fibrosis progression after Western diet feeding. (**A**) Staining of SD- or WD-fed mouse liver sections with desmin (scale bars: 50 µm), Sirius red, and GS (scale bars: 100 µm). Of note, GS expression expanded at week 36. In addition, central veins became localized to delicate fibrotic septa thus forming initial portal-central bridges indicating architectural distortion, which progressed until week 48. (**B**) Quantification of the desmin and Sirius red positive areas. Data represent mean and standard errors of 4–8 mice per time point. *: *p* < 0.05; **: *p* < 0.01; ***: *p* < 0.001 compared to SD week 3, Sidak’s multiple comparisons test; data of individual mice are illustrated by dots; SD: standard diet; WD: Western diet. (**C**) Whole slide scan (scale bar: 1000 µm) of a Sirius red-stained liver section from 48-week WD-fed mouse, with enlarged inset (scale bar: 30 µm) to show detail. (**D**) MRI analysis of the morphology, fat content, and hepatocyte uptake capacity after 48 weeks of SD and WD. (**E**,**F**) Quantification of the MRI signals representing fat content and hepatocyte uptake capacity. Data in E and F were acquired from three mice per time point; ***: *p* < 0.001 compared to SD, unpaired *t* test.

To study the time-dependent changes in fibrogenesis, we analyzed desmin- and Sirius red-stained sections. In the SD-fed mice, desmin staining was seen in the resident stellate cells along the sinusoids (Figure 6A). Conversely, progressive pericellular accumulation of desmin positive stellate cells was observed in the liver of WD-fed mice at week 18 and later (Figure 6A,B), which also co-localized with the DR (Figure S8). Similar to desmin, Sirius red staining revealed an accumulation of pericellular collagen which increased between weeks 18 and 48 after WD feeding (Figure 6A–C; Figure S8). The diffuse pericellular fibrosis formed initially in the midzonal/periportal zone as visualized by Sirius red and glutamine synthetase (GS) co-staining (Figure 6A), and later connections between the portal zones appeared but real fibrotic streaks free of hepatocytes were not observed as a dominant feature (Figure 6C). Fibrosis hinders the delivery of drugs to the hepatocyte sinusoidal membrane; thus, it negatively correlates with the hepatocyte uptake capacity [23]. We non-invasively quantified the functional impact of fibrosis on the hepatocyte uptake capacity using gadoxetic acid-enhanced MRI. First, fat content of the liver was determined in 48-week WD- and SD-fed mice by acquiring coronal images using a 3D multi-echo gradient-echo Dixon pulse sequence. Fat content of approximately 33% was detected in the livers of the WD-fed mice compared to only ~2.5% in the controls (Figure 6D,E). Next, to quantify the hepatocyte uptake capacity, T1-maps were acquired before, as well as one hour after, gadoxetic acid injection, and the difference (DT1) was calculated. Interestingly, DT1 reduced from ~93% in SD-fed mice to 79% in WD-fed mice (Figure 6D,F). In summary, long-term WD feeding led to progressive pericellular fibrosis, whose onset was preceded by the development of a functional bile-draining DR, which further progressed to a communicating chicken-wire type of fibrosis.

### 3.5. Reorganization of Zonally Expressed Enzymes and Functional Consequences

**Figure 7:**
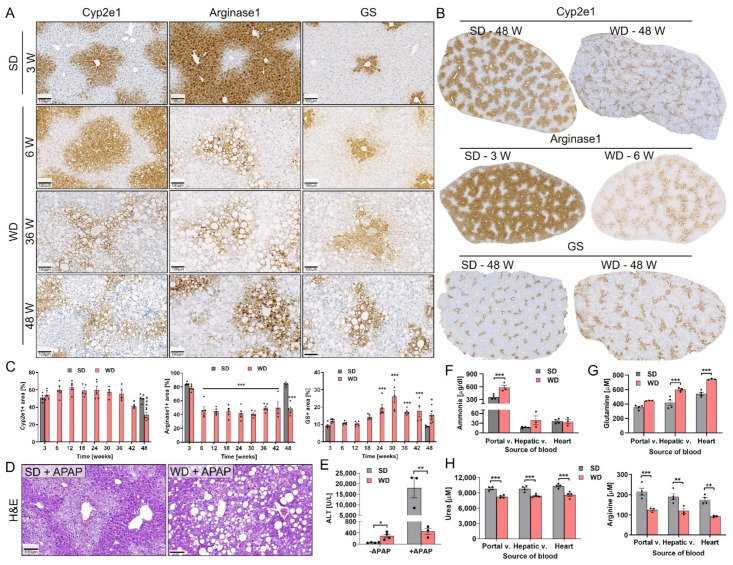
Lobular reorganization of metabolizing enzymes and functional consequences. (**A**) Reorganization of the pericentral enzymes Cyp2e1 and GS and the periportal/midzonal enzyme arginase1 in liver of WD-fed mice. (**B**) Decrease in Cyp2e1 (pericentral) and the arginase1 (periportal/midzonal) lobular regions and increase of the pericentral GS positive zone after WD feeding. (**C**) Quantification of the Cyp2e1, arginase1, and GS positive lobular areas in whole slide scans; data represent mean and standard errors of 3–8 mice per time point. *: *p* < 0.05; ***: *p* < 0.001 compared to SD week 3, Sidak’s multiple comparisons test; data of individual mice are illustrated by dots. (**D**,**E**) Hepatotoxicity of 300 mg/kg APAP in mice fed a SD or a WD for ~50 weeks as evidenced by H&E staining (**D**) and liver enzymes in blood (**E**); data represent mean and standard error of 3–4 mice per group. *: *p* < 0.05; **: *p* < 0.01 compared to SD, Tukey’s multiple comparisons test; data of individual mice are illustrated by dots. (**F**–**H**) Functional consequences of WD feeding (42 weeks) on ammonia (**F**), glutamine (**G**), urea and arginine (**H**) blood concentrations; data represent mean and standard error of 3–4 mice per group. **: *p* < 0.01; ***: *p* < 0.001 compared to SD, Sidak’s multiple comparisons test; data of individual mice are illustrated by dots. SD: standard diet; WD: Western diet; GS: glutamine synthetase; ALT: alanine transaminase. Scale bars: 100 µm.

The results of the previous chapters show that long-term feeding of mice on a WD recapitulates various stages of NAFLD progression. Therefore, in a next step we characterized the functional organization of the liver lobule during NASH progression. For this purpose, the spatial expression of the pericentral markers Cyp2e1 and GS, as well as the periportal marker arginase1, were analyzed using immunohistochemistry. This analysis revealed a reduction in the Cyp2e1 positive area in the WD-fed mice at weeks 42 and 48 compared to the SD-fed mice (Figure 7A–C). The area of the periportal urea cycle enzyme arginase1 also decreased, starting as early as week 6 of WD feeding (Figure 7A–C). In contrast, the GS positive area increased after WD feeding, particularly at week 24 and later (Figure 7A–C). Co-staining of GS and arginase1 showed complementary expression in the SD-fed mice. In contrast, although the GS positive area increased in the WD-fed mice, a GS and arginase1 negative zone was still observed (Figure S9).

Based on this altered zonation, we next studied the possible functional consequences. APAP toxicity was analyzed as a functional readout for Cyp2e1, since it metabolically activates APAP to the hepatotoxic NAPQI [44]. For this purpose, an overdose of APAP (300 mg/kg b.w.) was administered intraperitoneally to mice fed a WD or SD for 48 weeks. The expected APAP-induced pericentral damage was observed in the SD, but not in the WD group (Figure 7D). In agreement, there was a strong increase in blood transaminase activities in the SD-fed group, compared to the minor increase in the WD-fed mice (Figure 7E; Figure S10).

To study the functional consequences of the altered expression of GS and the urea cycle enzyme arginase1, concentrations of ammonia, and other related metabolites, such as glutamine and urea were analyzed in blood collected from the portal and hepatic veins, as well as from the right heart chamber of mice fed a SD or WD for 42 weeks. Ammonia concentrations were significantly elevated in the portal vein blood of the WD-fed mice (Figure 7F). Consistent with the elevated GS expression, glutamine concentrations were also significantly elevated in the hepatic vein and heart blood of the WD-fed mice, but not in the portal vein blood (Figure 7G). Blood concentrations of glutamate and the other amino acids, as well as the tricarboxylic acid cycle intermediates and metabolites were also significantly elevated in the WD-fed mice (Figure S11). In contrast, a significant reduction in urea and arginine levels was recorded at all measured positions in the WD-fed mice in comparison to the SD controls (Figure 7H), which agrees with the reduction in expression of the urea cycle enzymes.

Altogether, NASH progression is associated with altered expression of zonally expressed enzymes which has functional (and clinically relevant) consequences as indicated by the changes of urea cycle metabolites in blood and reduced cytochrome P450 metabolism.

### 3.6. Comparison of Key Histological Features of WD-Fed Mice to NAFLD Patients

**Figure 8:**
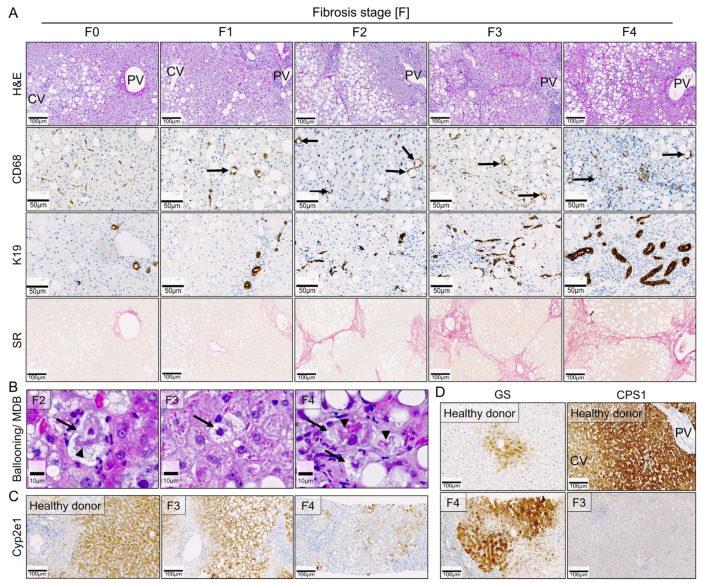
Comparison of the key features identified in the Western diet mice to NAFLD patients. (**A**) H&E staining (scale bars: 100 µm), lipogranulomas (CD68; scale bars: 50 µm) as identified with arrows, ductular reaction (K19; scale bars: 50 µm), and Sirius red staining (SR; scale bars: 100 µm) in NAFLD patients of all fibrosis stages (F0–F4). (**B**) Hepatocellular ballooning (arrows) and Mallory–Denk bodies (arrowheads, MDB) in F2–F4 patients; scale bars: 10 µm. (**C**) Reduced Cyp2e1 expression in F3 and F4 patients. Note, the lobular reorganization as highlighted by patchy periportal Cyp2e1 expression in F4 patients; scale bars: 100 µm. (**D**) Increase an abnormal periseptal localization of glutamine synthetase (GS) expression and decrease in carbamoyl-phosphate synthase 1 (CPS1) in NAFLD liver tissue with advanced fibrosis; scale bars: 100 µm. Abbreviations: CV, central vein; PV, portal vein; F3: fibrosis stage 3; F4: fibrosis stage 4.

To compare the above-described observations in the WD-fed mice to the human situation, a set of liver biopsies from 40 adult patients with NAFLD and known fibrosis stage (F0–F4) was used. Similar to our mouse model, H&E-stained liver tissue sections from the NAFLD patients revealed an accumulation of large LD (macrovesicular steatosis) in hepatocytes (Figure 8A); however, these LD initially appeared in the pericentral zone. Lipogranulomas (visualized by CD68 staining) were already identified in F1 patients, as well as in the more advanced stages (Figure 8A). Furthermore, progressive DR was observed in all NAFLD patients as determined by K19 staining (Figure 8A). Moreover, Sirius red staining showed diffuse pericellular fibrosis; however, fibrosis in these adult patients occurred pericentral, in contrast to the midzonal to periportal localization in mice (Figure 8A). Hepatocyte ballooning and Mallory–Denk bodies were detected, particularly in F2 and more advanced patients (Figure 8B). Interestingly, a strong decrease in Cyp2e1 expression was only detected in cirrhotic NAFLD patients (Figure 8C), which corresponds to the observation in the WD-fed mice, where Cyp2e1 loss was a late event. Similar to the WD-fed mice, an increase in GS expression was also seen in NAFLD patients (Figure 8D). This was accompanied by a strong reduction in the urea cycle enzyme CPS1 (Figure 8D).

In conclusion, the WD-fed mice recapitulate many features of human NAFLD, including macrovesicular steatosis, lobular inflammation and formation lipogranulomas, hepatocellular ballooning, formation of Mallory–Denk bodies, progressive fibrosis with DR, as well as metabolic reorganization of the hepatic lobules.

## 4. Discussion

Time-resolved analysis of the here-described mouse model of NAFLD led to the identification of a sequence of key events from bland steatosis to hepatocellular carcinoma formation (graphical abstract). Within the first three weeks of WD feeding, LD formed in the midzonal as well as the periportal regions of the liver lobules and increased in size and number up to week 12 when a plateau was reached. Reorganization of zonally expressed enzymes already began at week 6 when the initial periportal plus midzonal expression of the urea cycle enzyme arginase1 was restricted to a narrow margin of the most periportal hepatocytes. Furthermore, the pericentral cytochrome P450 expression was decreased, although this occurred only after longer periods on the WD. In contrast to the decline in expression of CYPs, the pericentral GS positive zone expanded strongly. Thus, WD feeding induced a complex reorganization of zonally expressed enzymes in the liver lobule. A functional consequence of decreased Cyp2e1 expression is the resistance to hepatotoxic doses of APAP which is metabolically activated in the pericentral zone of normal livers [44]. Consistent with this observation, APAP resistance was also described in other mouse models of chronic liver diseases, such as repeated CCl_4_ intoxication [27].

Zonal reorganization also led to complex changes in ammonia metabolism. The observed decrease in arginine and urea concentrations in the blood of WD-fed mice can be explained by reduced expression of urea cycle enzymes; this is consistent with a previous report of urea cycle dysregulation in NAFLD patients [45]. Furthermore, the expansion of the pericentral GS positive zone may explain the rise in glutamine concentrations in the liver vein and the systemic blood [24,46,47]. The observation that ammonia was neither elevated in the hepatic vein nor in the systemic blood suggests that the expansion of the GS positive zone compensates for the decrease in the region expressing urea cycle enzymes. Elevated ammonia in the portal vein blood can be explained by the increased glutamine blood concentrations, since glutamine is known to be consumed by the intestinal mucosa where it is metabolized by glutaminase to produce large quantities of ammonia, which is then reabsorbed into the portal vein [48,49].

Two types of inflammatory lesions were observed in the livers of Western diet-fed mice, namely, inflammatory foci and lipogranulomas, that represent well-known histological features of human NASH [41]. Intravital imaging of lipogranulomas revealed two types of lipogranulomas: type 1 that surrounds a viable steatotic hepatocyte, and type 2, where macrophages engulf the remaining LD of dead hepatocytes. Such a differentiation is virtually impossible ex vivo using fixed tissues. The present results are in agreement with previous studies that suggest a causal role for lipogranulomas in the progression of bland steatosis to steatohepatitis [50]. Of note, lipogranulomas are currently not considered during scoring of human NAFLD [41]. The observation that viable hepatocytes become enclosed by macrophages suggests that the macrophages do not only play a role in the removal of dead hepatocytes but may also contribute to hepatocyte death. The existence of type 2 lipogranulomas may be explained by an impaired phagocytosis of large LD, which may require longer periods of time.

Quantification of both inflammatory lesions showed a time course of initially very low inflammatory foci (week 3–30) and lipogranulomas (week 6–12). In this period, only mild increase in hepatocyte death and liver enzymes was observed. However, hepatocyte death and liver enzyme activities increased strongly together with the increase in lipogranulomas after week 12 and was accompanied by replacement proliferation. Increased levels of MLKL protein suggest that necroptosis is the responsible death mechanism [51].

Ductular reaction (DR) represents the next key event. A small but significant increase in DR, as detected by K19 immunostaining, was first observed at week 12 in the livers of the WD-fed mice, progressed over time and was associated with fibrotic remodeling of the hepatic lobules. The functional relevance of DR is currently controversially discussed. They may either serve to replenish lost hepatocytes in a setting of decompensated/impaired hepatocellular proliferation [43,52,53] or may represent an adaptive response to cholestasis, which may be an underappreciated feature of NAFLD [54,55]. Here, a recently established functional intravital imaging technique of bile acid transport was applied to investigate the role of DR [56,57]. Interestingly, intravenous administration of a green-fluorescent bile acid analogue resulted in the appearance of green fluorescence in the DR within a few minutes. This suggests that the DR is part of a functional bile-draining tubular network that is anatomically connected to the biliary tract, as similarly reported by Kamimoto et al. [55].

Fibrosis was associated with DR progression but started as focal pericellular collagen fiber deposition at week 18 and then progressed over time. At week 48 of WD feeding, reduced hepatocyte uptake capacity of gadoxetic acid from sinusoidal blood was observed, which may be due to the pericellular fibrosis. These results correspond to a previous study demonstrating that gadoxetic acid relative enhancement was lower in patients with NASH than in patients with simple steatosis [58].

After week 30, 59% of the WD-fed mice spontaneously developed tumors. The non-invasive identification of mice bearing tumors was reliably performed using MRI with a liver-specific contrast agent. Histological analyses revealed round-shaped, less proliferating, GS and K18 positive well-differentiated HCC nodules, and also irregularly formed, hyperproliferating, GS and K18 negative poorly differentiated HCCs. GS positive liver tumors in mice were reported to contain activating mutations of CTNNB1 and to exhibit several features of pericentral hepatocytes, while GS negative tumors carry activating mutations of Ha-ras or B-raf, have features similar to periportal hepatocytes, and were reported to be of no relevance with respect to human HCC [59]. Both the GS negative and the GS positive tumors found in our NAFLD model were negative for the periportal marker arginase1.

The above-described sequence of clearly distinguishable key events suggests that each of the eight processes may be accompanied by an orchestrated transcriptomic signature. However, it was surprising that the transcriptomic landscape was not in chronological correspondence to the key histopathological and functional events. Most gene expression changes occurred up to week 6. This is reflected by the two largest clusters, which were monotonously downregulated (cluster 1) or upregulated (cluster 7) and reached a plateau around week 12. Downregulated genes were associated with metabolism and further mature liver functions, while the upregulated genes were associated with immune functions. The only non-monotonous cluster (no. 5) showed peaks of gene expression changes at week 6 and 36 with lower levels at the intermediate periods (weeks 12–30). Week 6 represents the beginning of zonal reorganization, while week 36 coincides with tumor formation. It therefore may appear plausible that both key events are associated with major changes in the proteome, such as the degradation of pericentral and/or periportal proteins during zonal reorganization; but it should be considered that with only 21 genes, the non-monotonous cluster 5 is relatively small.

To study if genes that coincide with the key events ‘lipogranuloma formation’ and ‘fibrosis’ can be identified, we searched for rest-and-jump genes (RJG), defined as genes that were initially unaltered and only became deregulated after a specific period of WD feeding. Indeed, RJG genes were identified that were solely upregulated at weeks 18 and 24 of WD feeding, and therefore coincided with the formation of lipogranulomas and the onset of fibrosis. These genes included the *IL-21* receptor (*Il21r*) that plays a role in macrophage activation [60]; *Ccl5* which is known to be induced at later stages of inflammation compared to most other CC chemokines, and has been reported to recruit activated and memory T cells [61]; the caspase recruitment domain family signaling scaffold protein *Card11* that plays a not yet fully understood role in adaptive immunity and lymphocyte activation [62]; *Fam83a* whose function is not yet understood but expression has been shown to negatively correlate with tumor-infiltrating lymphocytes [63]; the surface glycoprotein *Cd8a*, a well-established marker of CD8^+^ T cells that has been shown to be elevated in an obese model of NASH and were reported to activate stellate cells [64]; the inhibitory T-cell immunoreceptor *Tigit*, also known to be expressed on NK cells [65]; and the cell surface glycoprotein *Scube1*, a marker of platelet activation and endothelial cell inflammation [66]. Together, these observations fit with previous reports that specific subsets of T cells create a microenvironment that activates macrophages to a more proinflammatory state [67], thereby contributing to the formation of lipogranulomas and necroptosis of hepatocytes. Despite their conspicuous course, it should be considered that RJG genes represent only a minority of all deregulated genes, and that the transcriptomic landscape is dominated by the patterns of clusters 1 and 7 with monotonous upregulation or downregulation of genes over time followed by a plateau.

The information on the sequence of key events will facilitate studies in future to identify relevant therapeutic targets. For example, it will be of interest if antagonization of preceding events will ameliorate later events, e.g., will antagonization of inflammatory foci or lipogranulomas reduce HCC; or will interventions that target ductular reaction ameliorate the progression of fibrosis? Moreover, the transcriptomics data will support the identification of genetic targets of translational relevance.

An important question addressed in the present study is to which degree the WD mouse model resembles human NAFLD, and to identify aspects that are different (Table 6). We confirm previous studies reporting that long-term WD-fed mice recapitulate key features of progressing human NAFLD, such as steatosis, lobular inflammation, hepatocyte ballooning, fibrosis, and HCC development [14], as well as DR and metabolic reorganization. However, an interspecies difference was observed with respect to zonation of steatosis. More specifically, LD and the subsequent events in mice initially developed in the midzonal/periportal lobular compartment. In contrast, LD predominantly formed in the pericentral region in human NAFLD in adult patients, but periportal fatty change has been observed in pediatric NAFLD [41,68]. In addition, there seem to be differences in terms of fibrosis progression with lack of central-to-portal and central-to-central septa in WD mice. The here-applied comparison of gene expression between humans and mice suggests that at least 30% of the genes with altered expression in human NAFLD are also differentially expressed in the WD mouse model, and that there is a substantial overlap with human HCC, of which the occurrence increases for the longer feeding periods. It should be considered that this technique of quantifying interspecies differences by recall and precision is conservative, because it is based on identically annotated genes. This means that even if they are related between species, genes do not contribute to the overlap if they carry different gene symbols. However, even the present conservative analysis shows that the WD mouse model recapitulates a substantial fraction of expression changes of human NAFLD.

One limitation of the present study is that similar to most studies in this field only male mice were studied. In future, it will be of interest to additionally analyze female mice and how the differences to males relate to the human situation.

In conclusion, the present spatio-temporal, multiscale study identified a sequence of translationally relevant key events in WD-fed mice developing steatohepatitis and HCC, which will support the identification of therapeutic targets in future.

## Figures and Tables

**Table 1:** Materials and resources.

Reagent or Resource	Source	Identifier
**Antibodies, Reagents, and Dyes Used for Immunohistochemistry**		
Anti-liver arginase1 antibody, rabbit	Abcam, Cambridge, UK	ab203490
Anti-arginase1 antibody, goat	Novus Biologicals, Littleton, USA	NB100-59740
Bodipy 495/503	Thermo Fisher Scientific, Waltham, USA	D3922
Anti-mouse CD45 antibody, rat	BD Bioscience, Heidelberg, Germany	550539
Anti-human CD68 monoclonal antibody, mouse	DakoCytomation A/S, Glostrup, Denmark	M0876
Anti-K18 polyclonal antibody, rabbit	Proteintech, Manchester, UK	10830-1-AP
Recombinant anti-K19 antibody, rabbit	Abcam, Cambridge, UK	ab52625
Recombinant anti-CPS1 monoclonal antibody, rabbit	Abcam, Cambridge, UK	ab129076
Anti-Cyp2e1 antibody, rabbit	Sigma-Aldrich, St. Louis, USA	HPA009128
Anti-mouse desmin antibody, rabbit	Thermo Fisher Scientific, Waltham, USA	RB -9014-P0
Anti-mouse F4/80 monoclonal antibody, rat	Bio-Rad, Hercules, USA	MCA497
Anti-GS polyclonal antibody, rabbit	Sigma, St. Louis, USA	G2781
Anti-GS polyclonal antibody, rabbit	Sigma, St. Louis, USA	G2781
Anti-Ki67 antibody, rabbit	Cell Signaling Technology, Danvers, USA	D3B5
Anti- cl. Caspase 3 (rabbit) monoclonal	Cell Signaling Technology, Danvers, USA	9661S
**Fluorescent Markers/Dyes Used for Intravital Imaging**		
Hoechst 33258	Thermo Fisher Scientific, Waltham, USA	H21491
Tetramethylrhodamine ethyl ester (TMRE)	Thermo Fisher Scientific, Waltham, USA	T669
Cholyl-lysyl-fluorescein (CLF)	Corning	451041
PE-F4/80 antibody	Thermo Scientific (eBioscience) , Waltham, USA	12-4801-82
Rhodamine 123	Thermo Fisher Scientific, Waltham, USA	R302
Bodipy 493/503	Thermo Fisher Scientific, Waltham, USA	D3922
**Mouse Diets**		
ssniff R/M-H, 10 mm standard diet	Ssniff, Soest, Germany	V1534-000
Western-style diet	Research Diets, New Brunswick, USA	D09100301
**Drugs/Contrast Agents/Toxins**		
Acetaminophen	Sigma-Aldrich, St. Louis, USA	A7085-500G
LPS	Sigma-Aldrich, St. Louis, USA	297-473-0
Gadoxetic acid (Primovist) 0.25 mmol/mL	Bayer, Wuppertal, Germany	KT07561
**Commercial Kits/Reagents**		
DeadEnd™ Fluorometric TUNEL System	Promega, Walldorf, Germany	G3250
Bluing Reagent	Roche, Mannheim, Germany	05 266 769 001
Discovery yellow Kit (RUO)	Roche, Mannheim, Germany	07 698 445 001
Discovery Teal HRP Kit (RUO)	Roche, Mannheim, Germany	8254338001
Chromo Map DAB	Roche, Mannheim, Germany	05 266 645 001
Piccolo general chemistry 13	Hitado, Möhnesee, Germany	AB-114-400-0029
Picrosirius Red Stain Kit	Polysciences Polysciences Inc., Warrington, USA	24901
RNeasy Mini Kit	Qiagen, Hilden, Germany	74116
RNase-Free DNase Set	Qiagen, Hilden, Germany	79254
RNA BR Assay Kit	Thermo Fisher Scientific, Waltham, USA	Q10210
RNA 6000 Nano Kit	Agilent Technologies, CA, USA	5067-1511
Qubit 1X dsDNA HS Assay Kit	Thermo Fisher Scientific, Waltham, USA	Q33230
DNA 1000 Kit	Agilent Technologies, CA, USA	5067-1504
**Antibodies Used for Western Blotting**		
Anti- MLKL	Biorbyt LLC, Cambridge, UK	orb32399
Anti- cleaved-Caspase-3	Cell Signaling Technology, Danvers, USA	9661S
Anti- GAPDH	AbD Serotec, Hercules, USA	MCA 4739
**Software and Algorithms**		
GraphPad Prism 9.1 Software	GraphPad, San Diego, USA	NA
Zen	Carl-Zeiss, Jena, Germany	NA
ImageJ 1.8.0_172	https://imagej.nih.gov; 22 April 2021	NA
**Instruments**		
LSM MP7 two-photon microscope	Zeiss, Jena, Germany	NA
Axio Scan.Z1	Zeiss, Jena, Germany	N/A
Confocal Laser Scanning Microscope FLUOVIEW FV1000	Olympus, Hamburg, Germany	N/A
DISCOVERY ULTRA Automated Slide Preparation System	Roche, Mannheim, Germany	N/A
Piccolo Xpress^®^ chemistry analyzer	Abaxis, Union City, USA	N/A
PocketChem BA PA-4140 ammonia meter	Arkray.inc, Amstelveen, The Netherlands	N/A
3Tesla MRI scanner	Prisma, Siemens Healthineers, Erlangen, Germany	

**Table 2:** Ingredients of the used Western-style diet.

Ingredient	Grams	Kcal	%
Casein, 80 mesh	200	800	22.12
l-cystine	3	12	0.33
Maltodextrin 10	100	400	11.06
Fructose	200	800	22.12
Sucrose	96	384	10.62
Cellulose, BW200	50	0	5.53
Soybean oil	25	225	2.77
Primex shortening	135	1215	14.93
Lard	20	180	2.21
Mineral Mix S10026	10	0	1.11
Dicalcium phosphate	13	0	1.44
Calcium carbonate	5.5	0	0.61
Potassium citrate, 1 H2O	16.5	0	1.83
Vitamin Mix V10001	10	40	1.11
Choline bitartrate	2	0	0.22
Cholesterol	18	0	1.99
FD&C Yellow dye	0.05	0	0.006
Total	904.05	4056	100
Total protein		20 Kcal %	22
Total carbohydrate		40 Kcal %	45
Total fat		40 Kcal %	20

**Table 3:** Overview of the number of analyzed mice per experimental condition.

Diet	Feeding Time (Weeks)	Number of Mice Analyzed								
		Liver/Body Weight, RNA-seq, Histology, IHC	HCC		MRI	Intravital Imaging	Nitrogen Metabolism	APAP Experiment	Necroptosis Analysis	LPS
			+	−						
**Western Diet**	3	5	0	5		3			4	
	6	5	0	5					4	
	9					3			-	
	12	5	0	5					4	
	18	5	0	5					4	
	24	5	0	5		6			4	
	30	5	4	1					4	
	32					3				
	36	5	1	4					4	
	42	4	5	4			3			
	48	8	6	2	4					3
	~50							7		
**Standard Diet**	3	7	0	7		3			4	
	6	5	0	5						
	30	5	0	5						
	36	7	0	7					4	
	42	3	0	3			4			
	48	5	0	5	4					
	~50							7		

**Table 4:** Fluorescent markers and functional dyes used for intravital imaging.

Dye/Marker	Marker for	Dose (mg/kg)	Vehicle	Two-Photon Excitation Range (nm)
Hoechst 33258	Nuclei	5	PBS	700–800
TMRE	Lobular zonation; mitochondrial membrane potential	0.96	Methanol/PBS (1:1)	780–820
Rhodamine123		0.8	Methanol/PBS (1:1)	720–820
Cholyl-lysyl-fluorescein	Bile acid analogue	1	PBS	740–820
Bodipy 493/503	Lipids	0.004	DMSO	900–940
PE-F4/80 antibody	Macrophages	0.06	PBS	720–760

**Table 5:** Antibodies/dyes used for immunohistochemistry analysis.

Target	Primary Antibodies		Secondary Antibodies	
	Antibody	Dilution	Antibody	Dilution
Lipids	Bodipy 495/503	2 µg/mL	-	-
Arginase1	Anti-arginase1 antibody, goat	1:100	Cy™5-conjugated AffiniPure donkey anti-goat IgG (H + L)	1:200
	Anti-liver arginase1 antibody, rabbit	1:2000	Ultra-Map anti-rabbit HRP	Automatic Discovery Ready to use
			Ultra-Map anti-rabbit alkaline phosphatase	
Leukocyte common antigen	Anti-mouse CD45 antibody, rat	1:400	Ultra-Map anti-rat HRP	
Macrophages, human	Anti-human CD68 monoclonal antibody, mouse	1:500	Ultra-Map anti-mouse HRP	
Cytoskeleton	Anti-K18 polyclonal antibody, rabbit	1:400	Ultra-Map anti-rabbit HRP	
Cholangiocyte, mouse	Recombinant anti-K19 antibody, rabbit	1:500	Ultra-Map anti-rabbit HRP	
Cholangiocyte, human	Recombinant anti-K19 antibody, rabbit	1:2000	Ultra-Map anti-rabbit HRP	
Carbamoyl-Phosphate Synthase1	Recombinant anti-CPS1 monoclonal antibody, rabbit	1:500	Ultra-Map anti-rabbit HRP	
Cyp2e1	Anti-Cyp2e1 antibody, rabbit	1:100	Ultra-Map anti-rabbit HRP	
			Ultra-Map anti-rabbit alkaline phosphatase	
Hepatic stellate cells	Anti-mouse desmin antibody, rabbit	1:400	Ultra-Map anti-rabbit HRP	
Macrophages, mouse	Anti-mouse F4/80 monoclonal antibody, rat	1:50	Ultra-Map anti-rat HRP	
Glutamine synthetase, mouse	Anti-GS polyclonal antibody, rabbit	1:15,000	Ultra-Map anti-rabbit HRP	
Apoptosis	Anti- cl. Caspase 3 monoclonal antibody, rabbit	1:500	Ultra-Map anti-rabbit HRP	
Glutamine synthetase, human	Anti-GS polyclonal antibody, rabbit	1:5000	Ultra-Map anti-rabbit HRP	
Cell proliferation antigen	Anti-Ki-67 antibody, rabbit	1:100	Ultra-Map anti-rabbit HRP	

**Table 6:** Similarities and differences of the present NAFLD mouse model and human NAFLD, concerning the key features analyzed in the present study.

Similarities	Differences
➢ Macrovesicular steatosis ➢ Zonal reorganization: ➢ Reduction of the periportal/midzonal zone expressing urea cycle enzymes ➢ Increase of the GS positive pericentral zone ➢ Decrease of the Cyp2e1 positive pericentral zone ➢ Lipogranulomas ➢ Hepatocyte ballooning ➢ Ductular reaction ➢ Pericellular fibrosis ➢ Hepatocellular cancer (HCC)	➢ Zonation of lipid droplets and fibrosis: midzonal/periportal in mice; pericentral in adult humans ➢ Rate of HCC formation is higher in mice than humans ➢ Only ~30% of the genes altered in human NAFLD are also differentially expressed in the present mouse model ➢ Hepatocyte ballooning: late during disease progression in mice; early during disease progression in humans

## Data Availability

The data presented in this study are available on request from the corresponding author.

## Supplementary Materials

The following are available online at https://www.mdpi.com/article/10.3390/cells10102516/s1, Table S1: Patient characteristics. Datasheet S1: Transcriptomics data. Videos S1 and S2. Intravital visualization of lipid droplets using the lipid dye bodipy (green) at 9 (Video S1) and 30 (Video S2) weeks after western diet (WD) feeding. Differentiation of the periportal and the pericentral lobular zones was achieved using the mitochondrial dye TMRE that leads to a stronger signal in the periportal than the pericentral zone. Videos S3 and S4A. Intravital imaging of livers of WD-fed mice after intravenous injection of a fluorophore-coupled F4/80 antibody (red), the mitochondrial membrane potential marker Rhodamine123 and Hoechst for nuclear staining. Video S3. shows Kupffer cells (red) in the sinusoidal wall of a mouse fed on WD for 3 weeks. Video S4A. A WD-fed mouse for 32 weeks showing a vital steatotic hepatocyte with mitochondrial and nuclear structures surrounded by F4/80 positive macrophages (white circle), and a lipid droplet enclosed by macrophages without discernible mitochondria or nuclear signal (pink circle). Video S4B. Intravital imaging of the liver of a WD-fed mouse for 24 weeks after intravenous injection of the mitochondrial membrane potential marker TMRE (red), the lipid dye bodipy (green) and the nuclear dye Hoechst (blue), showing a lipid droplet enclosed by macrophages without discernible mitochondria or nuclear signal (circle). Figure S1. Body weight changes and liver-to-body weight ratio in mice after feeding on standard diet up to 48 weeks. Figure S2. No major alterations in liver tissue morphology and zonated enzyme expressions after 48-week standard diet (SD) feeding to mice. Figure S3. Early midzonal/periportal (weeks 3–6) and late pan lobular (week 30) distribution of lipid droplets after western diet (WD) feeding. Figure S4. Intravital visualization of lipid droplets using the lipid dye bodipy (green) at 9 and 30 weeks after western diet (WD) feeding. Figure S5. Hematoxylin and eosin staining of tumor and non-tumor tissue in 48-week western diet-fed mice. Figure S6. Non-invasive detection of tumors in 48-week western diet-fed mice using MRI. Figure S7. Transcriptomics data. Figure S8. Whole slide scans from the livers of standard diet- (SD) fed mice for 3 weeks and at different time intervals after western diet (WD) feeding showing the progression of ductular reaction (CK19 staining) and fibrosis (desmin and Sirius red staining). Figure S9. Co-staining of glutamine synthetase (GS) and arginase1 in the livers of standard (SD) and western diet (WD) fed mice. Figure S10. Hepatotoxicity of 300 mg/kg APAP in mice fed a SD or a WD for ~50 weeks as evidenced by aspartate transaminase (AST) activity in heart blood. Figure S11. Functional consequences of WD feeding (42 weeks) on amino acids and citric acid cycle intermediates as well as metabolites.

## Abbreviations

ALP: alkaline phosphatase; ALT: alanine transaminase; APAP: acetaminophen; AST: aspartate transaminase; b.w.: body weight; CLF: cholyl-lysyl-fluorescein; DR: ductular reaction; GS: glutamine synthetase; K-18/19: keratin-18/19; LD: lipid droplets; LPS: lipopolysaccharide; MDB: Mallory–Denk bodies; MLKL: mixed lineage kinase domain-like; MRI: magnetic resonance imaging; NAFLD: non-alcoholic fatty liver disease; NASH: non-alcoholic steatohepatitis; NAPQI: N-acetyl-p-benzoquinone imine; PC: pericentral; PV: periportal; R123: rhodamine123; RJG: rest-and-jump genes; SD: standard diet; TMRE: tetramethylrhodamine ethyl ester; WD: Western diet.

## References

[1] Estes C., Anstee Q.M., Arias-Loste M.T., Bantel H., Bellentani S., Caballeria J., Colombo M., Craxi A., Crespo J., Day C.P. (2018). Modeling NAFLD disease burden in China, France, Germany, Italy, Japan, Spain, United Kingdom, and United States for the period 2016–2030. J. Hepatol..

[2] Lazarus J.V., Ekstedt M., Marchesini G., Mullen J., Novak K., Pericas J.M., Roel E., Romero-Gómez M., Ratziu V., Tacke F. (2019). A cross-sectional study of the public health response to non-alcoholic fatty liver disease in Europe. J. Hepatol..

[3] Ramadori P., Weiskirchen R., Trebicka J., Streetz K. (2015). Mouse models of metabolic liver injury. Lab. Anim..

[4] Schuppan D., Schattenberg J.M. (2013). Non-alcoholic steatohepatitis: Pathogenesis and novel therapeutic approaches. J. Gastroenterol. Hepatol..

[5] Friedman S.L., Neuschwander-Tetri B.A., Rinella M., Sanyal A.J. (2018). Mechanisms of NAFLD development and therapeutic strategies. Nat. Med..

[6] Rinella M.E., Tacke F., Sanyal A.J., Anstee Q.M. (2019). Report on the AASLD/EASL joint workshop on clinical trial endpoints in NAFLD. J. Hepatol..

[7] Younossi Z., Anstee Q.M., Marietti M., Hardy T., Henry L., Eslam M., George J., Bugianesi E. (2017). Global burden of NAFLD and NASH: Trends, predictions, risk factors and prevention. Nat. Rev. Gastroenterol. Hepatol..

[8] Jahn D., Dorbath D., Schilling A.-K., Gildein L., Meier C., Vuille-Dit-Bille R., Schmitt J., Kraus D., Fleet J.C., Hermanns H.M. (2019). Intestinal vitamin D receptor modulates lipid metabolism, adipose tissue inflammation and liver steatosis in obese mice. Biochim. Biophys. Acta (BBA)—Mol. Basis Dis..

[9] Tsuchida T., Lee Y.A., Fujiwara N., Ybanez M., Allen B., Martins S., Fiel M.I., Goossens N., Chou H.-I., Hoshida Y. (2018). A simple diet- and chemical-induced murine NASH model with rapid progression of steatohepatitis, fibrosis and liver cancer. J. Hepatol..

[10] Drescher H., Weiskirchen R., Fülöp A., Hopf C., Román E.G.D.S., Huesgen P.F., De Bruin A., Bongiovanni L., Christ A., Tolba R. (2019). The Influence of Different Fat Sources on Steatohepatitis and Fibrosis Development in the Western Diet Mouse Model of Non-alcoholic Steatohepatitis (NASH). Front. Physiol..

[11] Farrell G., Schattenberg J.M., Leclercq I., Yeh M.M., Goldin R., Teoh N., Schuppan D. (2018). Mouse Models of Nonalcoholic Steatohepatitis: Toward Optimization of Their Relevance to Human Nonalcoholic Steatohepatitis. Hepatology.

[12] Machado M.V., Michelotti G.A., Xie G., De Almeida T.P., Boursier J., Bohnic B., Guy C.D., Diehl A.M. (2015). Mouse Models of Diet-Induced Nonalcoholic Steatohepatitis Reproduce the Heterogeneity of the Human Disease. PLoS ONE.

[13] Hansen H.H., Feigh M., Veidal S.S., Rigbolt K.T., Vrang N., Fosgerau K. (2017). Mouse models of nonalcoholic steatohepatitis in preclinical drug development. Drug Discov. Today.

[14] Asgharpour A., Cazanave S.C., Pacana T., Seneshaw M., Vincent R., Banini B.A., Kumar D.P., Daita K., Min H.-K., Mirshahi F. (2016). A diet-induced animal model of non-alcoholic fatty liver disease and hepatocellular cancer. J. Hepatol..

[15] Stephenson K., Kennedy L., Hargrove L., Demieville J., Thomson J., Alpini G., Francis H. (2018). Updates on Dietary Models of Nonalcoholic Fatty Liver Disease: Current Studies and Insights. Gene. Expr..

[16] Schuran F.A., Lommetz C., Steudter A., Ghallab A., Wieschendorf B., Schwinge D., Zuehlke S., Reinders J., Heeren J., Lohse A.W. (2021). Aryl Hydrocarbon Receptor Activity in Hepatocytes Sensitizes to Hyperacute Acetaminophen-Induced Hepatotoxicity in Mice. Cell. Mol. Gastroenterol. Hepatol..

[17] Schneider K.M., Elfers C., Ghallab A., Schneider C.V., Galvez E.J., Mohs A., Gui W., Candels L.S., Wirtz T.H., Zuehlke S. (2021). Intestinal Dysbiosis Amplifies Acetaminophen-Induced Acute Liver Injury. Cell. Mol. Gastroenterol. Hepatol..

[18] Campos G., Schmidt-Heck W., De Smedt J., Widera A., Ghallab A., Pütter L., González D., Edlund K., Cadenas C., Marchan R. (2020). Inflammation-associated suppression of metabolic gene networks in acute and chronic liver disease. Arch. Toxicol..

[19] Koeppert S., Ghallab A., Peglow S., Winkler C.F., Graeber S., Büscher A., Hengstler J.G., Jahnen-Dechent W. (2021). Live Imaging of Calciprotein Particle Clearance and Receptor Mediated Uptake: Role of Calciprotein Monomers. Front. Cell Dev. Biol..

[20] Reif R., Ghallab A., Beattie L., Günther G., Kuepfer L., Kaye P.M., Hengstler J.G. (2016). In vivo imaging of systemic transport and elimination of xenobiotics and endogenous molecules in mice. Arch. Toxicol..

[21] Ghallab A., Hassan R., Myllys M., Albrecht W., Friebel A., Hoehme S., Hofmann U., Seddek A.-L., Braeuning A., Kuepfer L. (2021). Subcellular spatio-temporal intravital kinetics of aflatoxin B1 and ochratoxin A in liver and kidney. Arch. Toxicol..

[22] Zhong X., Nickel M.D., Kannengiesser S.A., Dale B.M., Kiefer B., Bashir M.R. (2013). Liver fat quantification using a multi-step adaptive fitting approach with multi-echo GRE imaging. Magn. Reson. Med..

[23] Yoon J.H., Lee J.M., Kang H.-J., Ahn S.J., Yang H., Kim E., Okuaki T., Han J.K. (2019). Quantitative Assessment of Liver Function by Using Gadoxetic Acid–enhanced MRI: Hepatocyte Uptake Ratio. Radiology.

[24] Ghallab A., Cellière G., Henkel S.G., Driesch D., Hoehme S., Hofmann U., Zellmer S., Godoy P., Sachinidis A., Blaszkewicz M. (2015). Model-guided identification of a therapeutic strategy to reduce hyperammonemia in liver diseases. J. Hepatol..

[25] Schenk A., Ghallab A., Hofmann U., Hassan R., Schwarz M., Schuppert A., Schwen L.O., Braeuning A., Teutonico D., Hengstler J. (2017). Physiologically-based modelling in mice suggests an aggravated loss of clearance capacity after toxic liver damage. Sci. Rep..

[26] Holland C.H., Ramirez Flores R.O., Myllys M., Hassan R., Edlund K., Hofmann U., Marchan R., Cadenas C., Reinders J., Hoehme S. (2021). Transcriptomic cross-species analysis of chronic liver disease reveals consistent regulation between humans and mice. Hepatol. Commun..

[27] Ghallab A., Myllys M., Holland C.H., Zaza A., Murad W., Hassan R., Ahmed Y.A., Abbas T., Abdelrahim E.A., Schneider K.M. (2019). Influence of Liver Fibrosis on Lobular Zonation. Cells.

[28] Patro R., Duggal G., Love M.I., Irizarry M.I.L.R.A., Kingsford C. (2017). Salmon provides fast and bias-aware quantification of transcript expression. Nat. Methods.

[29] Love M.I., Soneson C., Hickey P.F., Johnson L.K., Pierce N.T., Shepherd L., Morgan M., Patro R. (2020). Tximeta: Reference sequence checksums for provenance identification in RNA-seq. PLoS Comput. Biol..

[30] Love M.I., Huber W., Anders S. (2014). Moderated estimation of fold change and dispersion for RNA-seq data with DESeq2. Genome. Biol..

[31] Young M.D., Wakefield M.J., Smyth G.K., Oshlack A. (2010). Gene ontology analysis for RNA-seq: Accounting for selection bias. Genome. Biol..

[32] Itzel T., Neubauer M., Ebert M., Evert M., Teufel A. (2020). Hepamine—A Liver Disease Microarray Database, Visualization Platform and Data-Mining Resource. Sci. Rep..

[33] Lian Q., Wang S., Zhang G., Wang D., Luo G., Tang J., Chen L., Gu J. (2018). HCCDB: A Database of Hepatocellular Carcinoma Expression Atlas. Genom. Proteom. Bioinform..

[34] Schneider A.T., Gautheron J., Feoktistova M., Roderburg C., Loosen S.H., Roy S., Benz F., Schemmer P., Büchler M.W., Nachbur U. (2016). RIPK1 Suppresses a TRAF2-Dependent Pathway to Liver Cancer. Cancer Cell.

[35] Hofmann U., Maier K., Niebel A., Vacun G., Reuss M., Mauch K. (2007). Identification of metabolic fluxes in hepatic cells from transient13C-labeling experiments: Part I. Experimental observations. Biotechnol. Bioeng..

[36] Maier K., Hofmann U., Reuss M., Mauch K. (2010). Dynamics and Control of the Central Carbon Metabolism in Hepatoma Cells. BMC Syst. Biol..

[37] Bankhead P., Loughrey M.B., Fernández J.A., Dombrowski Y., McArt D., Dunne P.D., McQuaid S., Gray R.T., Murray L.J., Coleman H.G. (2017). QuPath: Open source software for digital pathology image analysis. Sci. Rep..

[38] Berg S., Kutra D., Kroeger T., Straehle C.N., Kausler B.X., Haubold C., Schiegg M., Ales J., Beier T., Rudy M. (2019). ilastik: Interactive machine learning for (bio)image analysis. Nat. Methods..

[39] Rimola J., Forner A., Sapena V., Llarch N., Darnell A., Díaz A., García-Criado A., Bianchi L., Vilana R., Díaz-González A. (2019). Performance of gadoxetic acid MRI and diffusion-weighted imaging for the diagnosis of early recurrence of hepatocellular carcinoma. Eur. Radiol..

[40] Sutti S., Albano E. (2019). Adaptive immunity: An emerging player in the progression of NAFLD. Nat. Rev. Gastroenterol. Hepatol..

[41] Kleiner D.E., Brunt E.M., Van Natta M., Behling C., Contos M.J., Cummings O.W., Ferrell L.D., Liu Y.-C., Torbenson M.S., Unalp-Arida A. (2005). Design and validation of a histological scoring system for nonalcoholic fatty liver disease. Hepatology.

[42] Gadd V.L., Skoien R., Powell E., Fagan K.J., Winterford C., Horsfall L., Irvine K., Clouston A.D. (2014). The portal inflammatory infiltrate and ductular reaction in human nonalcoholic fatty liver disease. Hepatology.

[43] Schuppan D., Surabattula R., Wang X. (2018). Determinants of fibrosis progression and regression in NASH. J. Hepatol..

[44] Sezgin S., Hassan R., Zühlke S., Kuepfer L., Hengstler J.G., Spiteller M., Ghallab A. (2018). Spatio-temporal visualization of the distribution of acetaminophen as well as its metabolites and adducts in mouse livers by MALDI MSI. Arch. Toxicol..

[45] De Chiara F., Heebøll S., Marrone G., Montoliu C., Hamilton-Dutoit S., Ferrandez A., Andreola F., Rombouts K., Grønbæk H., Felipo V. (2018). Urea cycle dysregulation in non-alcoholic fatty liver disease. J. Hepatol..

[46] Bartl M., Pfaff M., Ghallab A., Driesch D., Henkel S.G., Hengstler J.G., Schuster S., Kaleta C., Gebhardt R., Zellmer S. (2015). Optimality in the zonation of ammonia detoxification in rodent liver. Arch. Toxicol..

[47] Schliess F., Hoehme S., Henkel S.G., Ghallab A., Driesch D., Böttger J., Guthke R., Pfaff M., Hengstler J.G., Gebhardt R. (2014). Integrated metabolic spatial-temporal model for the prediction of ammonia detoxification during liver damage and regeneration. Hepatology.

[48] Häussinger D., Rodes J.B.J., Blei A., Reichen J., Rizzetto M. (2007). Ammonia, urea production and pH regulation. Hepatology: From Basic Science to Clinical Practice.

[49] Hawkins R.A., Jessy J., Mans A.M., Chedid A., DeJoseph M.R. (1994). Neomycin Reduces the Intestinal Production of Ammonia from Glutamine. Adv. Exp. Med. Biol..

[50] Lefere S., Tacke F. (2019). Macrophages in obesity and non-alcoholic fatty liver disease: Crosstalk with metabolism. JHEP Rep..

[51] Schwabe R.F., Luedde T. (2018). Apoptosis and necroptosis in the liver: A matter of life and death. Nat. Rev. Gastroenterol. Hepatol..

[52] Manco R., Clerbaux L.-A., Verhulst S., Nader M.B., Sempoux C., Ambroise J., Bearzatto B., Gala J.L., Horsmans Y., van Grunsven L. (2019). Reactive cholangiocytes differentiate into proliferative hepatocytes with efficient DNA repair in mice with chronic liver injury. J. Hepatol..

[53] Raven A., Lu W.-Y., Man T.Y., Ferreira-Gonzalez S., O’Duibhir E., Dwyer B., Thomson J.P., Meehan R., Bogorad R., Koteliansky V. (2017). Cholangiocytes act as facultative liver stem cells during impaired hepatocyte regeneration. Nature.

[54] Jörs S., Jeliazkova P., Ringelhan M., Thalhammer J., Dürl S., Ferrer J., Sander M., Heikenwalder M., Schmid R.M., Siveke J.T. (2015). Lineage fate of ductular reactions in liver injury and carcinogenesis. J. Clin. Investig..

[55] Kamimoto K., Nakano Y., Kaneko K., Miyajima A., Itoh T. (2020). Multidimensional imaging of liver injury repair in mice reveals fundamental role of the ductular reaction. Commun. Biol..

[56] Ghallab A., Hofmann U., Sezgin S., Vartak N., Hassan R., Zaza A., Godoy P., Schneider K.M., Guenther G., Ahmed Y. (2018). Bile Microinfarcts in Cholestasis Are Initiated by Rupture of the Apical Hepatocyte Membrane and Cause Shunting of Bile to Sinusoidal Blood. Hepatology.

[57] Schneider K.M., Candels L.S., Hov J.R., Myllys M., Hassan R., Schneider C.V., Wahlström A., Mohs A., Zühlke S., Liao L. (2021). Gut microbiota depletion exacerbates cholestatic liver injury via loss of FXR signaling. Nat. Metab..

[58] Bastati N., Feier D., Wibmer A., Traussnigg S., Balassy C., Tamandl D., Einspieler H., Wrba F., Trauner M., Herold C. (2014). Noninvasive Differentiation of Simple Steatosis and Steatohepatitis by Using Gadoxetic Acid–enhanced MR Imaging in Patients with Nonalcoholic Fatty Liver Disease: A Proof-of-Concept Study. Radiology.

[59] Hailfinger S., Jaworski M., Braeuning A., Buchmann A., Schwarz M. (2006). Zonal gene expression in murine liver: Lessons from tumors. Hepatology.

[60] Pesce J., Kaviratne M., Ramalingam T.R., Thompson R.W., Urban J., Cheever A.W., Young D.A., Collins M., Grusby M.J., Wynn T.A. (2006). The IL-21 receptor augments Th2 effector function and alternative macrophage activation. J. Clin. Investig..

[61] Seo W., Shimizu K., Kojo S., Okeke A., Kohwi-Shigematsu T., Fujii S.-I., Taniuchi I. (2020). Runx-mediated regulation of CCL5 via antagonizing two enhancers influences immune cell function and anti-tumor immunity. Nat. Commun..

[62] Bedsaul J.R., Carter N., Deibel K.E., Hutcherson S.M., Jones T.A., Wang Z., Yang C., Yang Y.-K., Pomerantz J.L. (2018). Mechanisms of Regulated and Dysregulated CARD11 Signaling in Adaptive Immunity and Disease. Front. Immunol..

[63] Zhou F., Wang X., Liu F., Meng Q., Yu Y. (2020). FAM83A drives PD-L1 expression via ERK signaling and FAM83A/PD-L1 co-expression correlates with poor prognosis in lung adenocarcinoma. Int. J. Clin. Oncol..

[64] Breuer D.A., Pacheco M.C., Washington M.K., Montgomery S.A., Hasty A.H., Kennedy A. (2020). CD8+ T cells regulate liver injury in obesity-related nonalcoholic fatty liver disease. Am. J. Physiol. Liver Physiol..

[65] Ostroumov D., Duong S., Wingerath J., Woller N., Manns M.P., Timrott K., Kleine M., Ramackers W., Roessler S., Nahnsen S. (2020). Transcriptome Profiling Identifies TIGIT as a Marker of T-Cell Exhaustion in Liver Cancer. Hepatology.

[66] Liao W.-J., Wu M.-Y., Peng C.-C., Tung Y.-C., Yang R.-B. (2019). Epidermal growth factor-like repeats of SCUBE1 derived from platelets are critical for thrombus formation. Cardiovasc. Res..

[67] Van Herck M.A., Weyler J., Kwanten W.J., Dirinck E.L., De Winter B.Y., Francque S.M., Vonghia L. (2019). The Differential Roles of T Cells in Non-alcoholic Fatty Liver Disease and Obesity. Front. Immunol..

[68] Hudert C.A., Selinski S., Rudolph B., Bläker H., Loddenkemper C., Thielhorn R., Berndt N., Golka K., Cadenas C., Reinders J. (2018). Genetic determinants of steatosis and fibrosis progression in paediatric non-alcoholic fatty liver disease. Liver Int..

