# Supplementary material for: Spatio-Temporal Multiscale Analysis of Western Diet-Fed Mice Reveals a Translationally Relevant Sequence of Events during NAFLD Progression
Source: Cells. 2021 Sep 23;10(10):2516. doi: 10.3390/cells10102516 (PMC8533774; doi:10.3390/cells10102516)
Supplement: Supplementary file 1 [file cells-10-02516-s001.zip › Supplemental data/Suppl. Table S1.pdf]

Supplemental Table S1. Patient characteristics.

Patient	Fibrosis stage	Gender	Age [years]
1	F0	male	54
2		male	50
3		male	43
4		male	46
5		female	43
6		male	32
7		female	30
8	F1	female	49
9		female	44
10		female	45
11		female	19
12		male	35
13		male	41
14		female	48
15		male	54
16		male	52
17		male	68
18	F2	female	27
19		female	34
20		male	41
21		male	49
22		male	59
23		female	65
24		female	40
25	F3	male	41
26		male	67
27		male	64
28		male	61
29		male	56
30		female	69
31		male	56
32		female	59
33		male	56
34	F4	male	44
35		female	49
36		male	40
37		male	57
38		female	68
39		male	52
