# Supplementary material for: Spatio-Temporal Multiscale Analysis of Western Diet-Fed Mice Reveals a Translationally Relevant Sequence of Events during NAFLD Progression
Source: Cells. 2021 Sep 23;10(10):2516. doi: 10.3390/cells10102516 (PMC8533774; doi:10.3390/cells10102516)
Supplement: Supplementary file 1 [file cells-10-02516-s001.zip › Supplemental data/Supplemental data.pdf]

Supplemental materials to:

Spatio-temporal multiscale analysis of Western diet-fed mice reveals a translationally relevant sequence of events during NAFLD progression

Ahmed Ghallab, Maiju Myllys, Adrian Friebel, Julia Duda, Karolina Edlund, Emina Halibasic, Mihael Vucur, Zaynab Hobloss, Lisa Brackhagen, Brigitte Begher-Tibbe, Reham Hassan, Michael Burke, Erhan Genc, Lynn Johann Frohwein, Ute Hofmann, Christian H. Holland, Daniela Gonzalez, Magdalena Zak-Keller, Abdel-latif Seddek, Tahany Abbas, Elsayed S. I. Mohammed, Andreas Teufel, Timo Itzel, Sarah Metzler, Rosemarie Marchan, Cristina Cadenas, Carsten Watzl, Michael A. Nitsche, Franziska Kappenberg, Tom Luedde, Thomas Longerich, Jörg Rahnenführer, Stefan Hoehme, Michael Trauner, Jan G. Hengstler

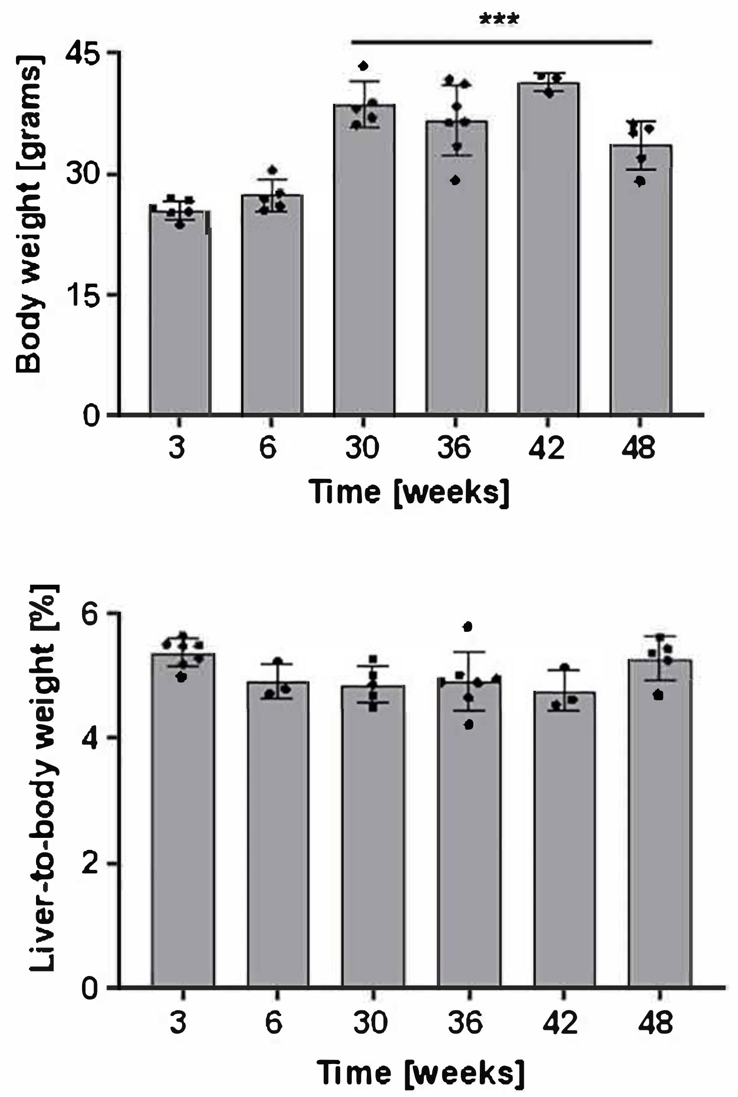

Suppl. Figure S1. Body weight changes and liver-to-body weight ratio in mice after feeding on standard diet up to 48 weeks. Data represent the mean and standard error of 3-7 mice per time point. ***: p<0.001 compared to SD week 3, Dunnett's multiple comparisons tests; data of individual mice are illustrated by dots.

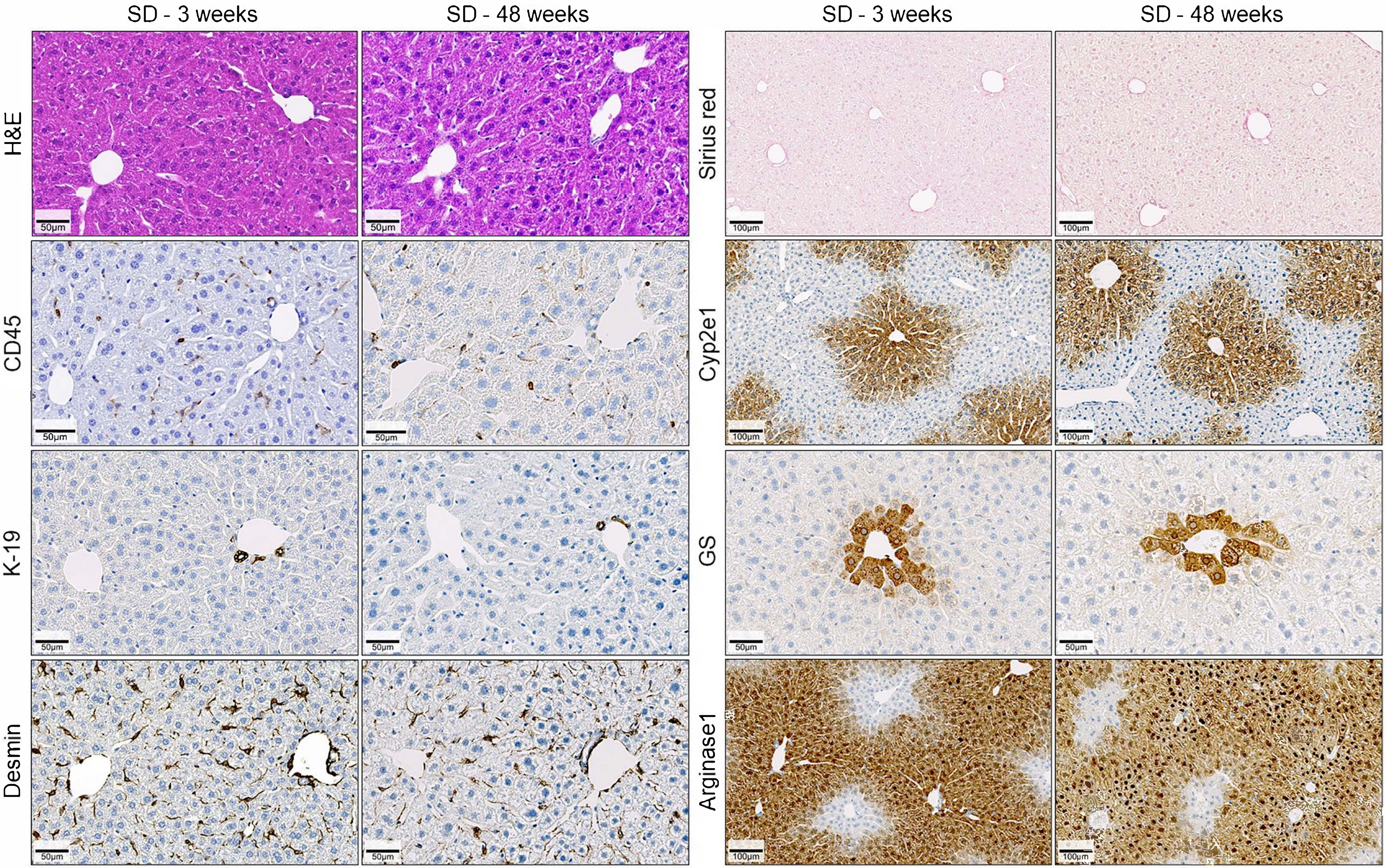

Suppl. Figure S2. No major alterations in liver tissue morphology and zonated enzyme expressions after 48-week standard diet (SD) feeding to mice.

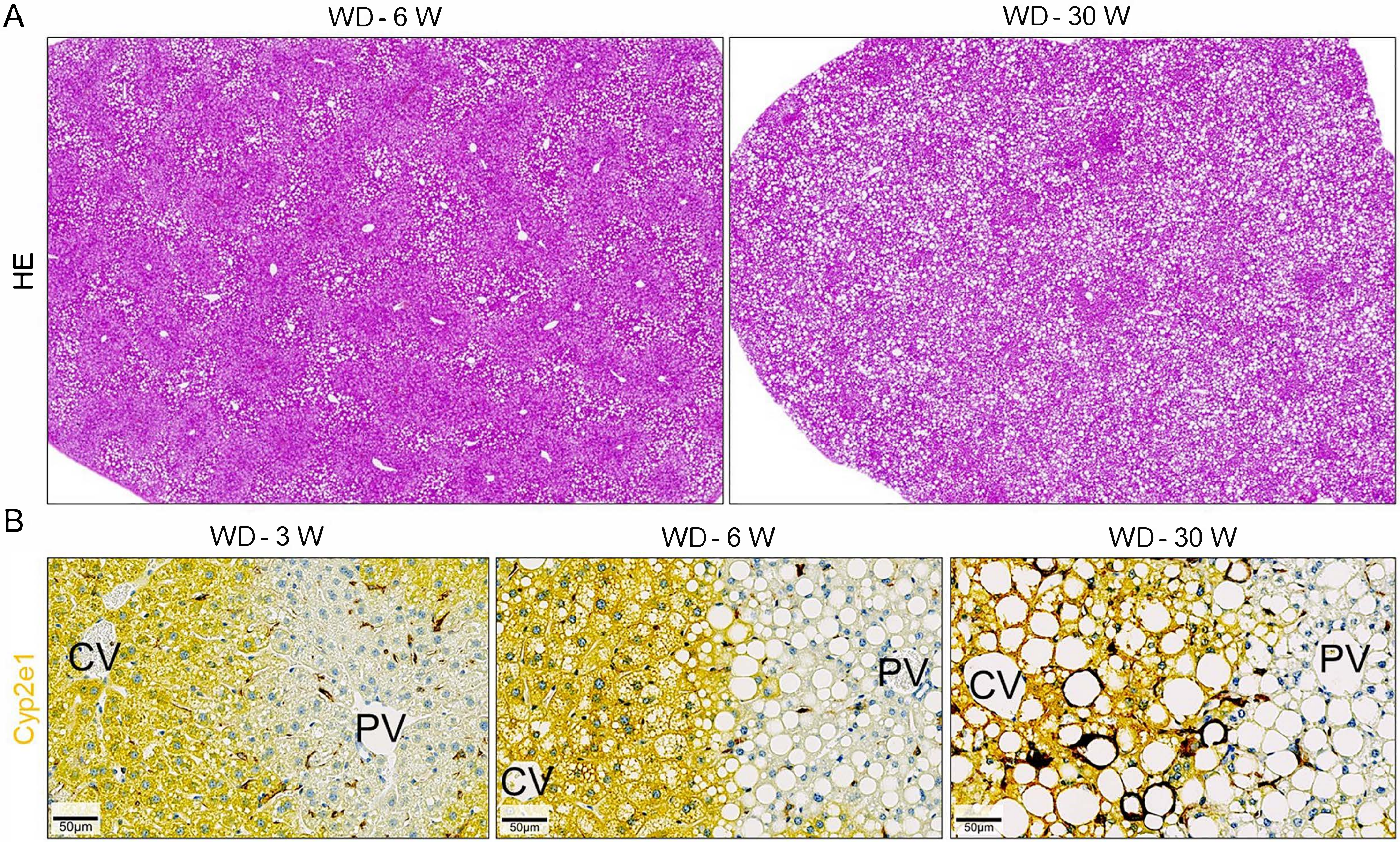

Suppl. Figure S3. Early midzonal/periportal (weeks 3-6) and late pan lobular (week 30) distribution of lipid droplets after western diet (WD) feeding. HE: hematoxylin and eosin; CV: central vein; PV: portal vein.

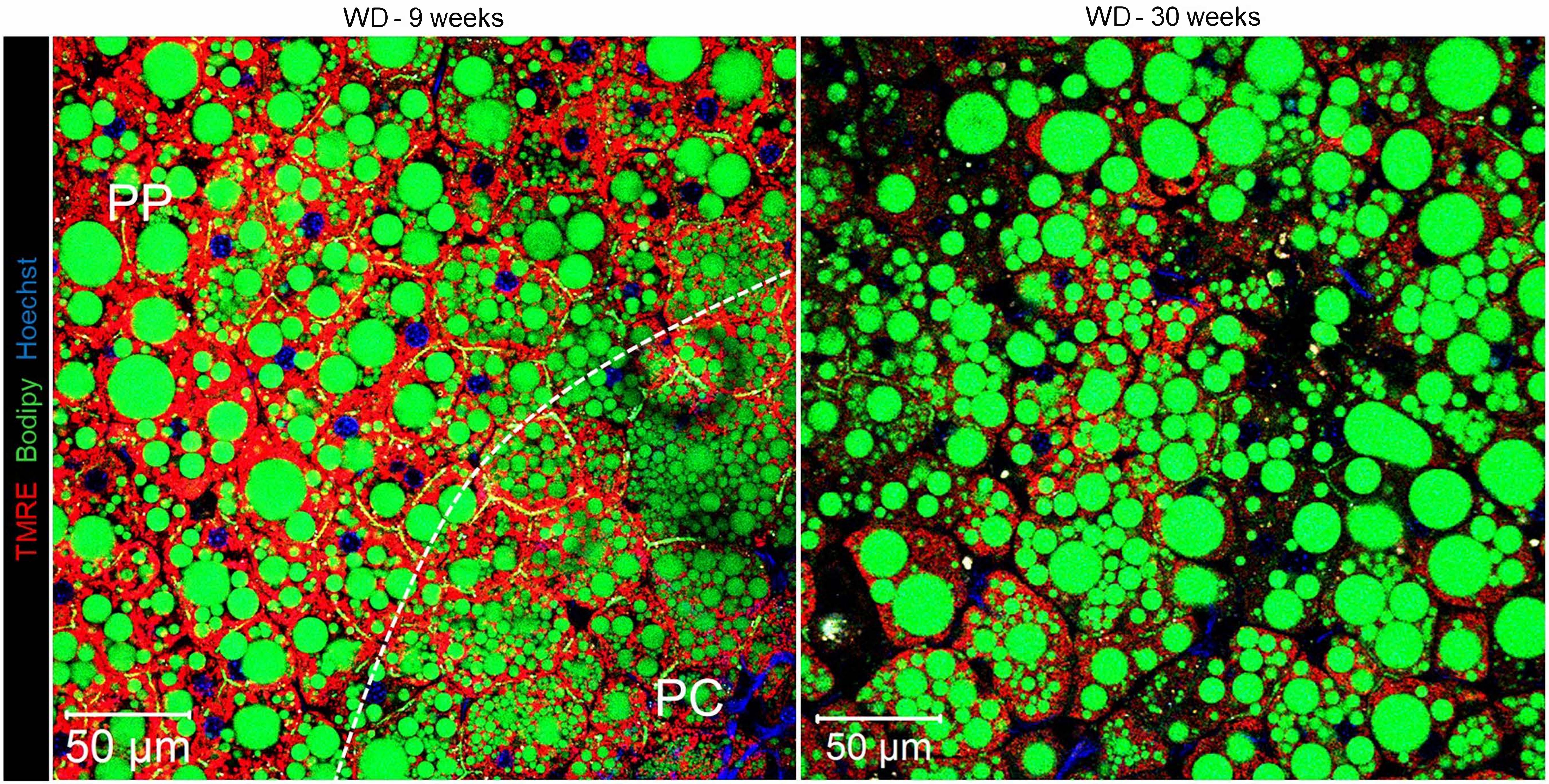

Suppl. Figure S4. Intravital visualization of lipid droplets using the lipid dye bodipy (green) at 9 and 30 weeks after western diet (WD) feeding. Differentiation of the periportal (PP) and the pericentral (PC) lobular zones was achieved using the mitochondrial dye TMRE that leads to a stronger signal in the PP than the PC zone.

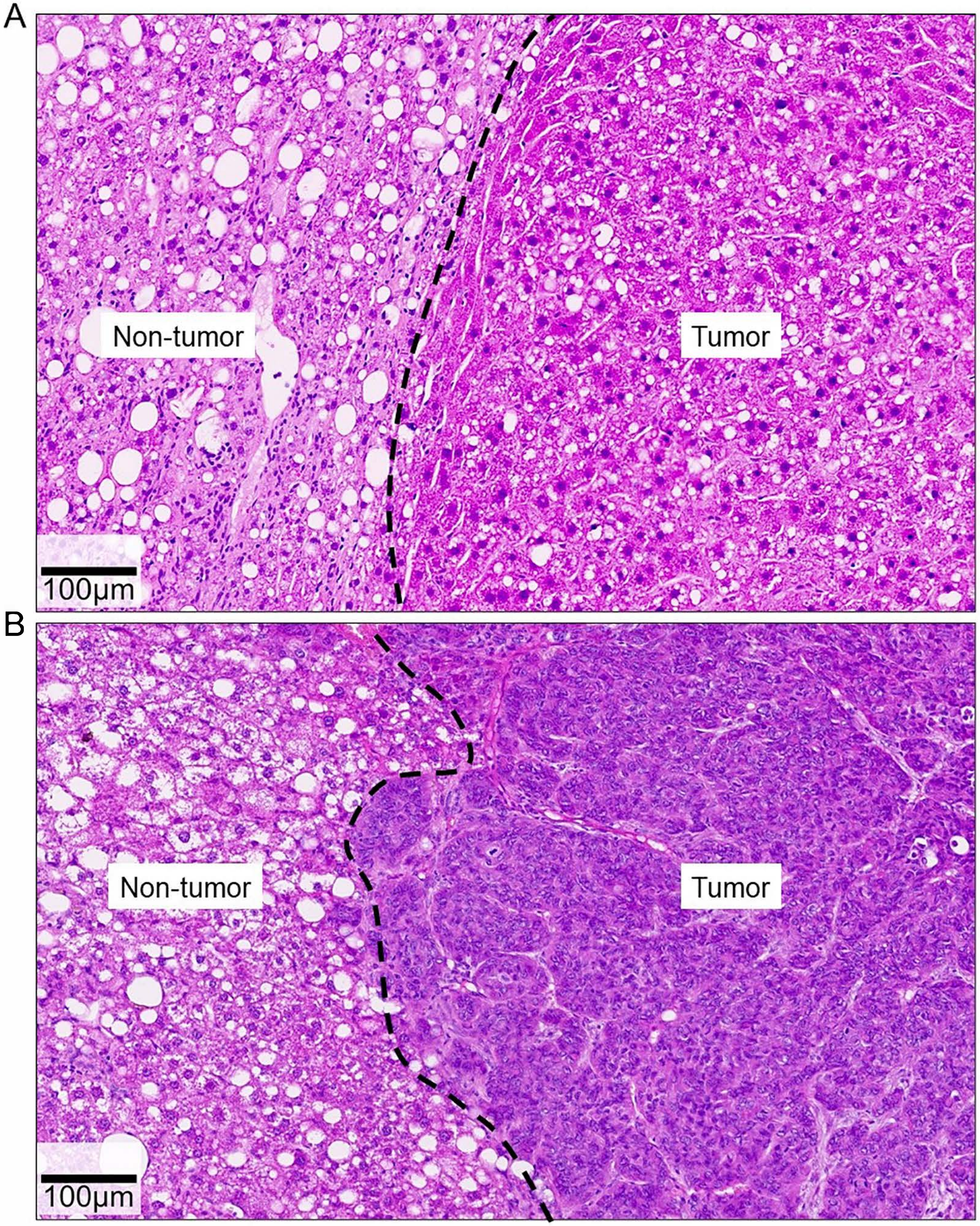

Suppl. Figure S5. Hematoxylin and eosin staining of tumor and non-tumor tissue in 48-week western diet-fed mice. **A.** A glutamine synthetase (GS) positive tumor consisting of cells which are slightly smaller in size and less steatotic compared to those in the surrounding non-tumor tissue. **B.** A GS negative tumor nodule consisting of cells which are very small in size and completely free of lipid droplets compared to the surrounding non-tumor tissue.

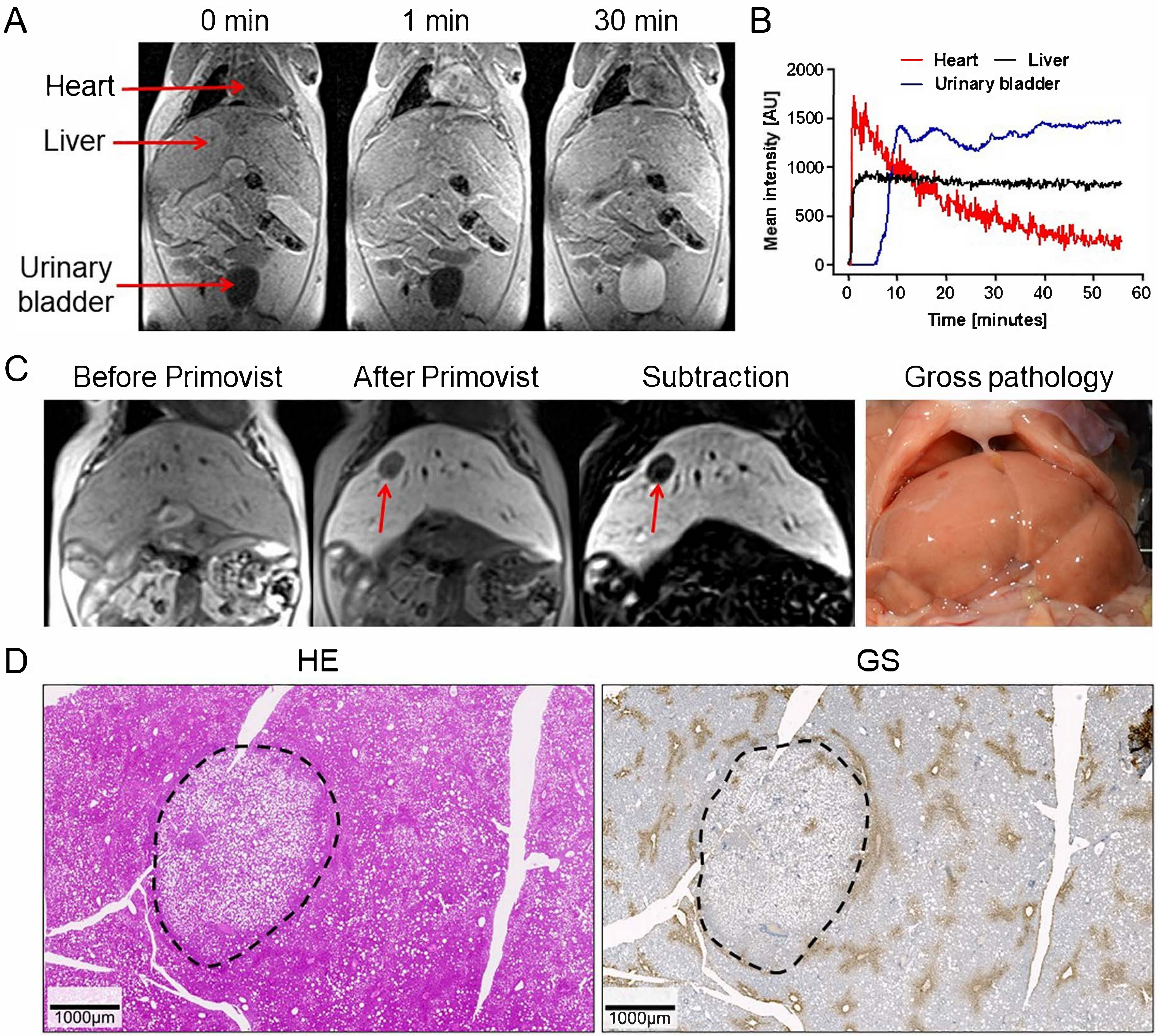

Suppl. Figure S6. Non-invasive detection of tumors in 48-week western diet-fed mice using MRI. **A.** Stills from MRI analysis of a WD-fed mouse without tumors before (0 minute), as well as 1 and 30 minutes after injection of the contrast agent Primovist which shows homogeneous enrichment in the liver parenchyma. **B.** Quantification of Primovist signals in the heart, liver parenchyma, and urinary bladder. **C.** Visualization of a tumor nodule that appears as a hypodense region (red arrows) in the liver of a WD-fed mouse for 48 weeks. The tumor nodule was non-detectable in the gross pathology but was clearly visible in histopathology (**D**).

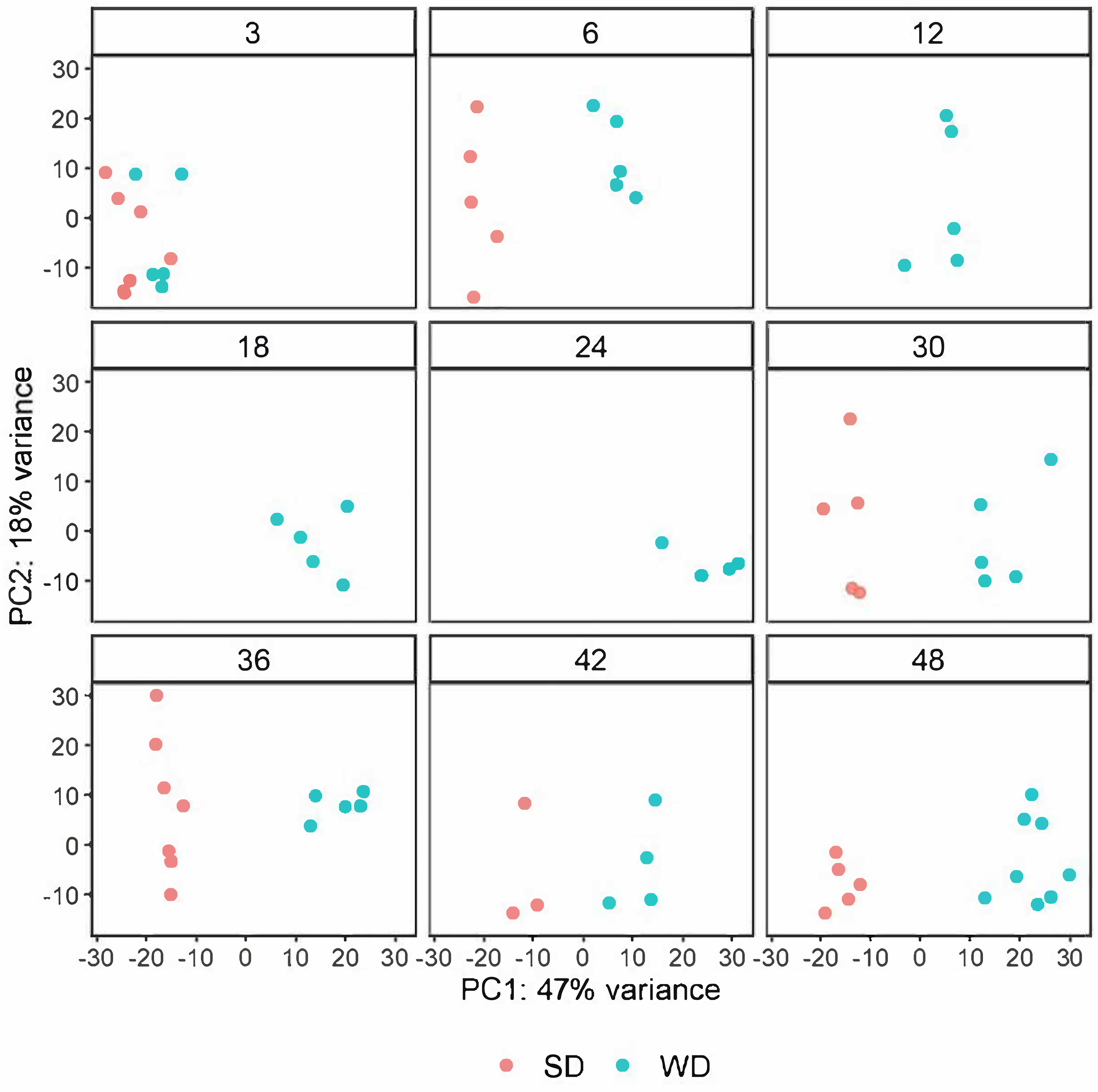

Suppl. Figure S7A. Principle component analysis (PCA) comparing mice fed a standard diet (SD) or a Western diet (WD) for 3 to 48 weeks. Each dot represents an individual mouse.

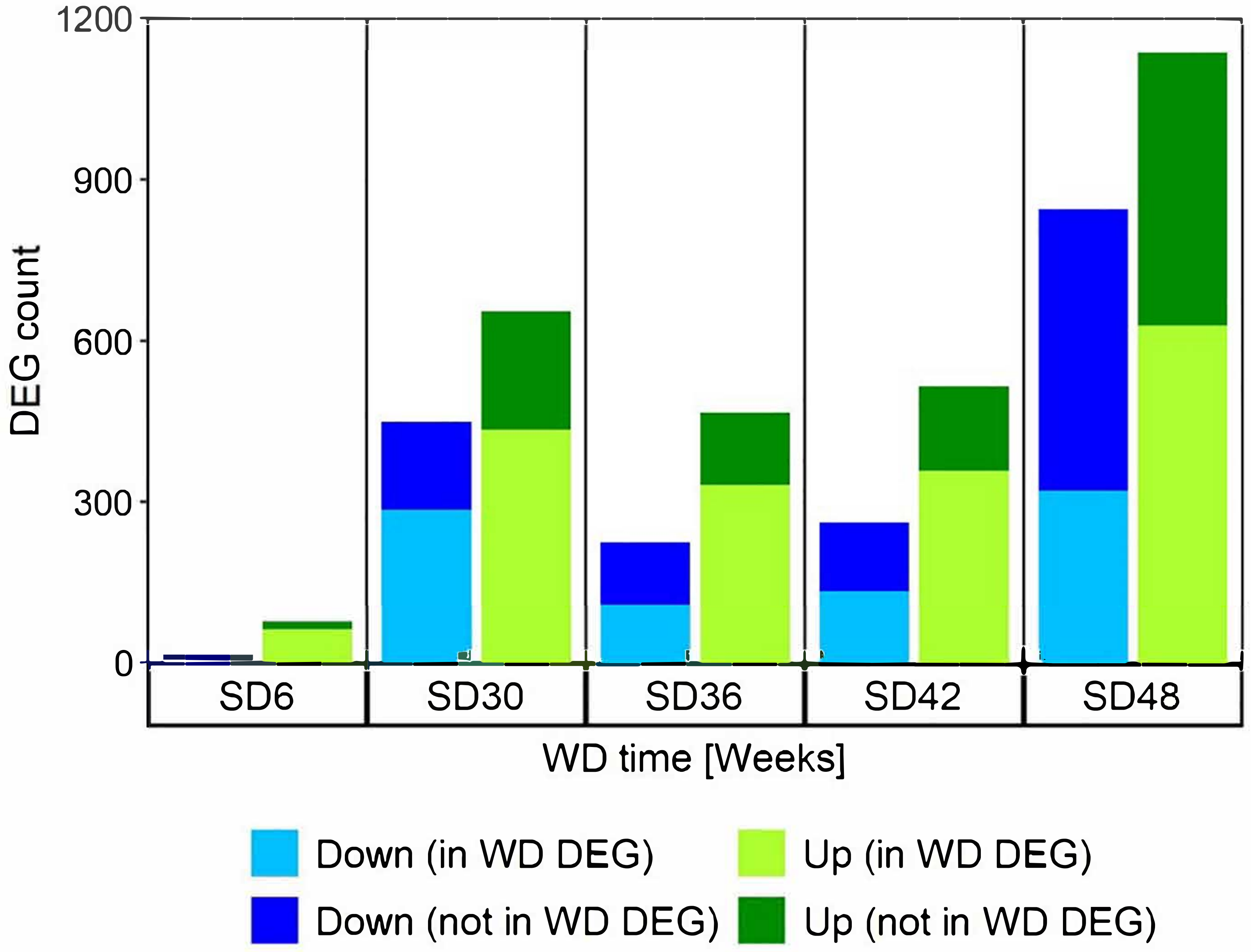

Suppl. Figure S7B. Numbers of differentially expressed genes (DEG) of mice fed a standard diet (SD) for 6, 30, 36, 42, and 48 weeks compared to SD week 3; adj p<0.01; abs($\log_2$ fold change) > $\log_2$ (1.5). The light blue and light green color indicate DEGs that are differentially expressed also in the WD Fed groups of the corresponding time periods.

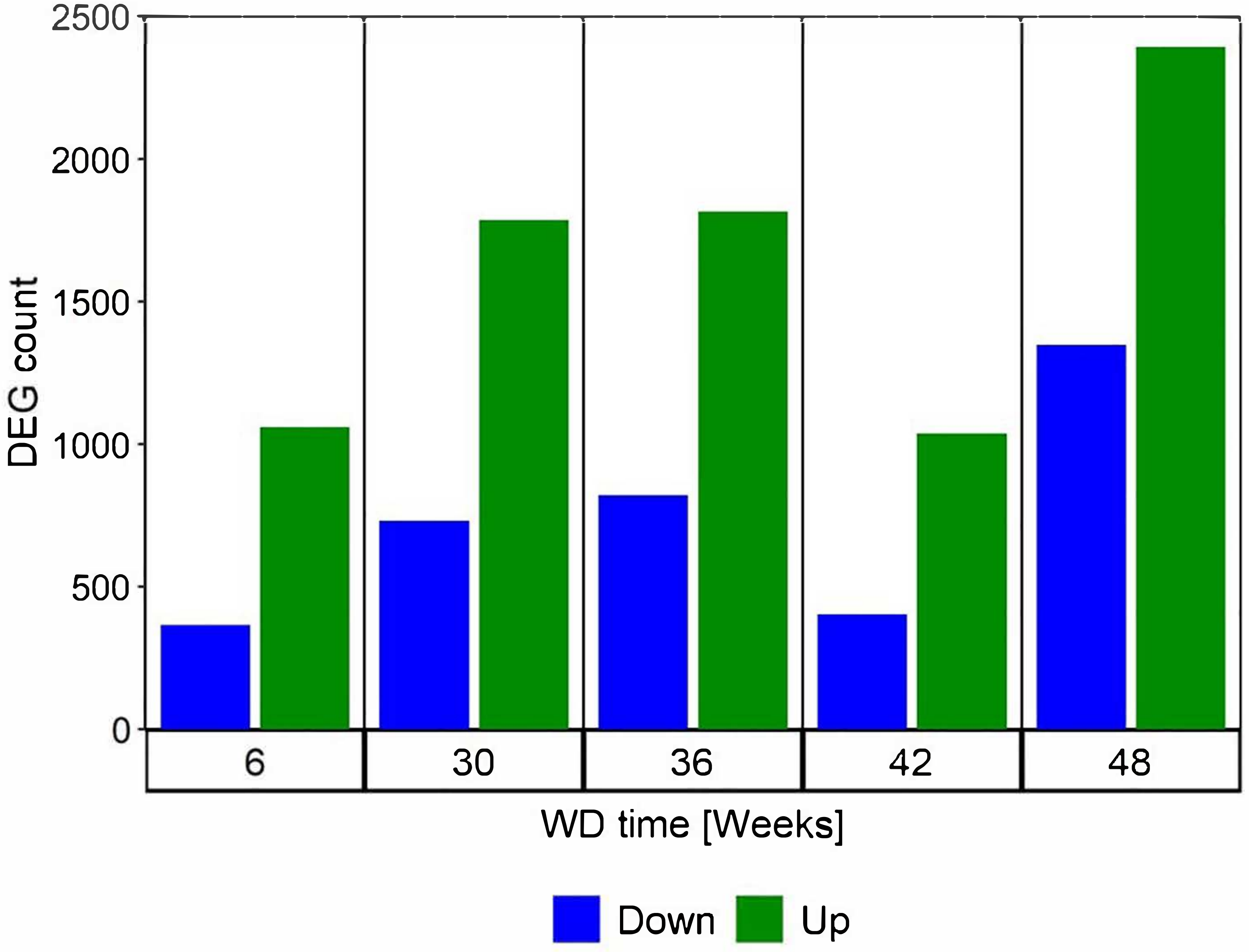

Suppl. Figure S7C. Numbers of differentially expressed genes (DEG) of mice fed a Western diet (WD) Compared to standard diet (SD) for the indicated time periods; adj p<0.01; abs($\log_2$ fold change) > $\log_2(1.5)$.

Suppl. Figure S7D. Complete set of ‘rest and jump genes‘ (RJG) of week 6. RJG week 6 remain unaltered until week 6 of WD compared to week 3 of SD and are up- or downregulated thereafter.

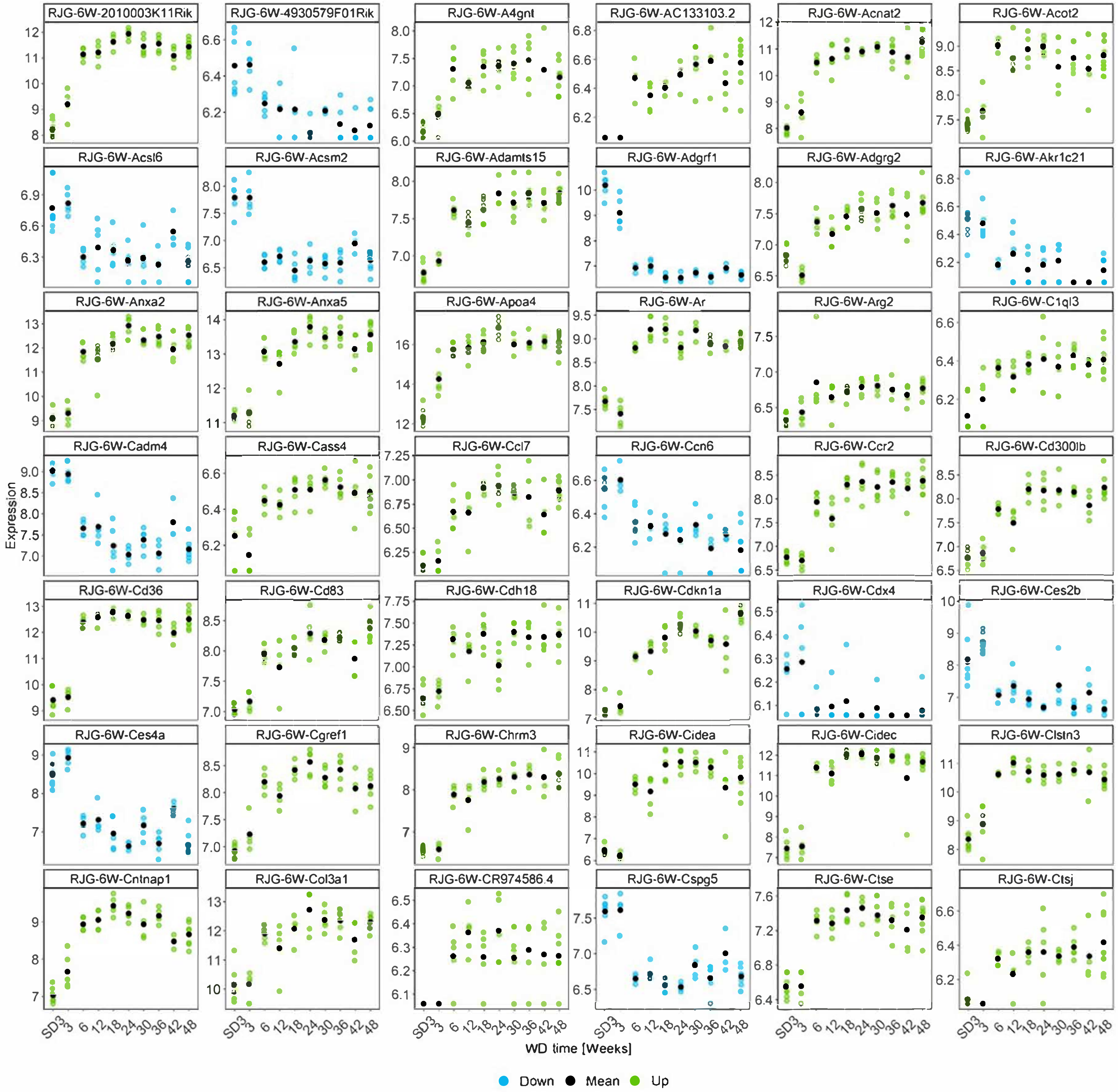

Suppl. Figure S7D.
Continued (part 2)

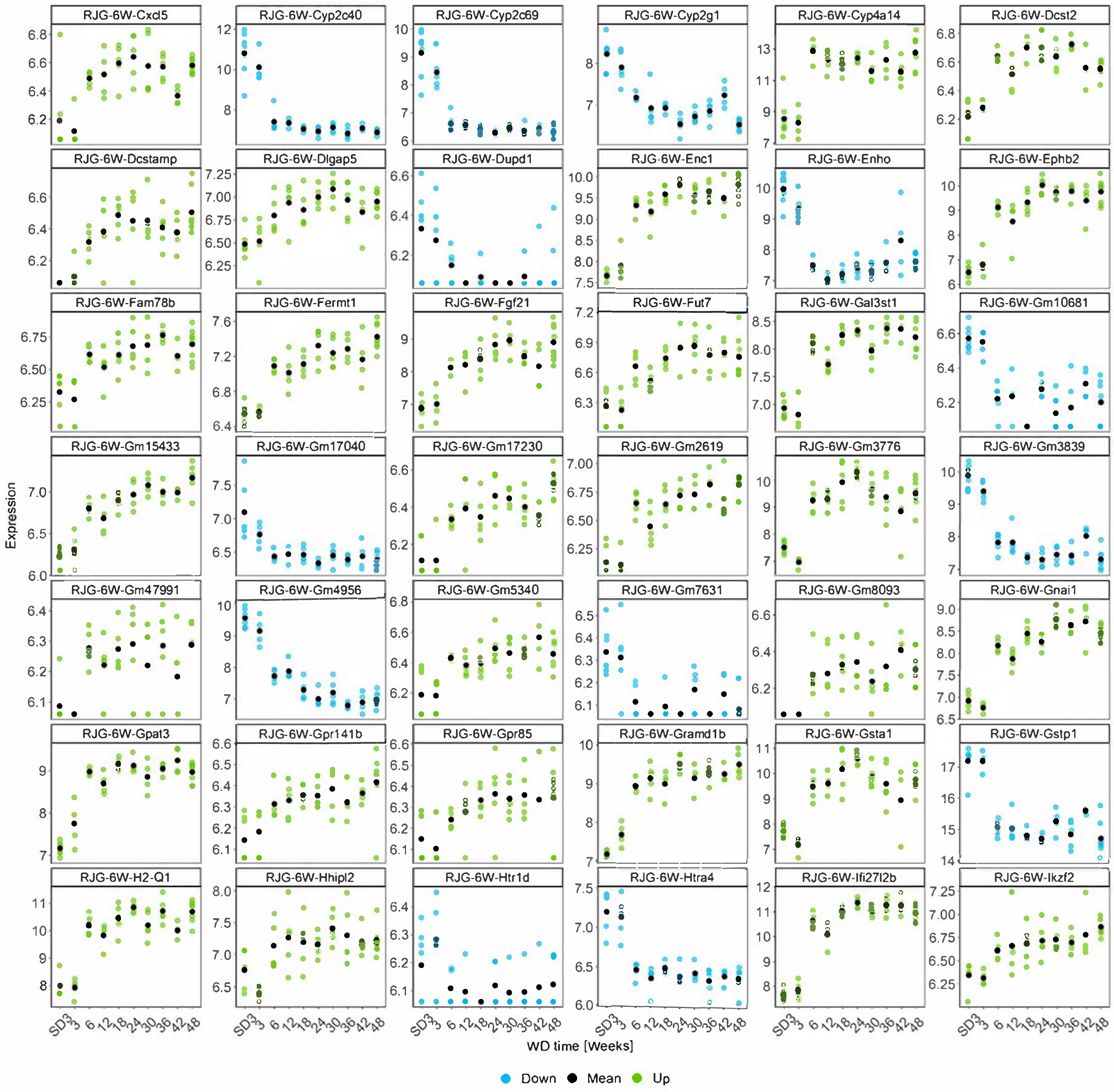

Suppl. Figure S7D.
Continued (part 3)

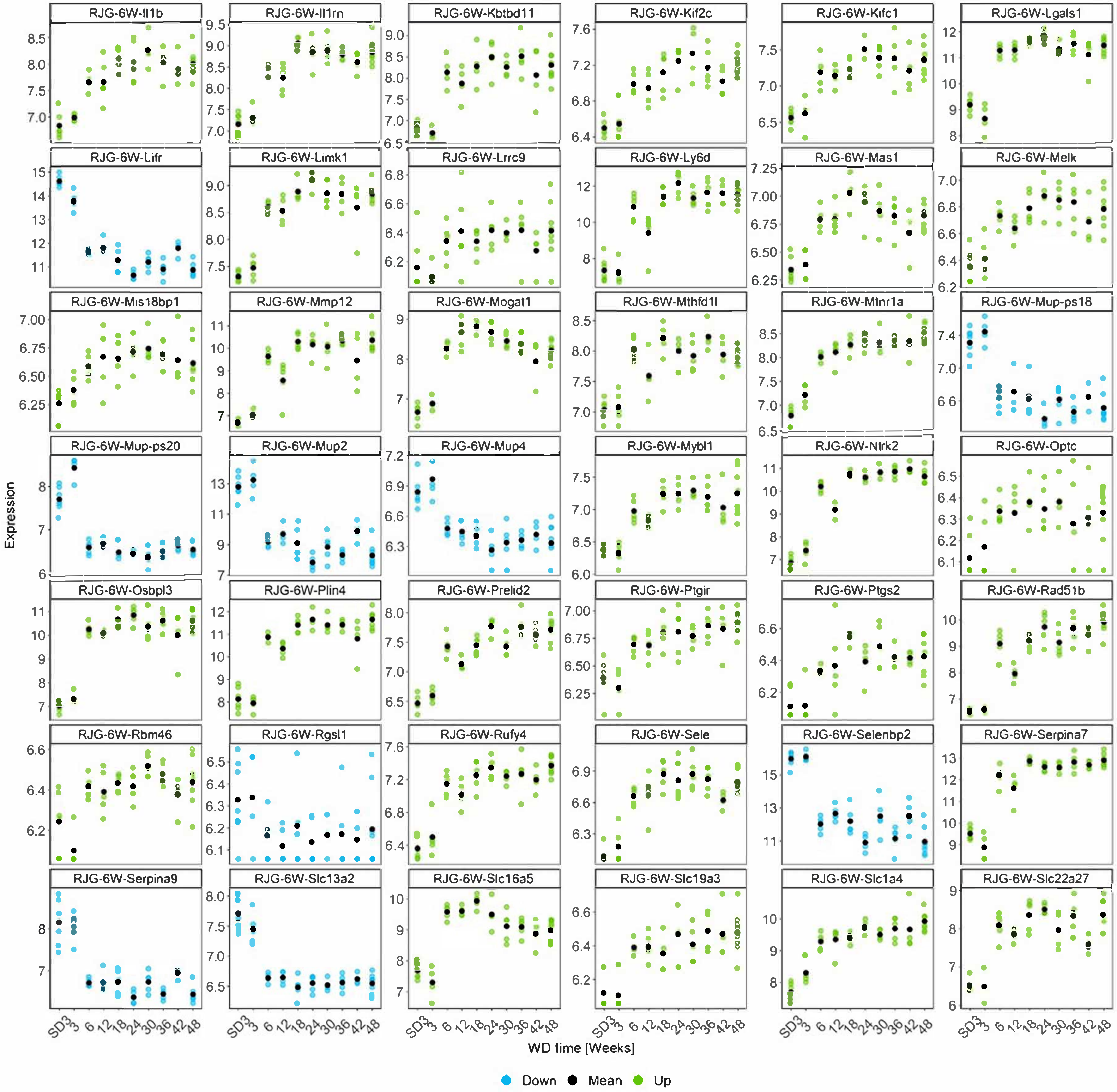

Suppl. Figure S7D.
Continued (part 4)

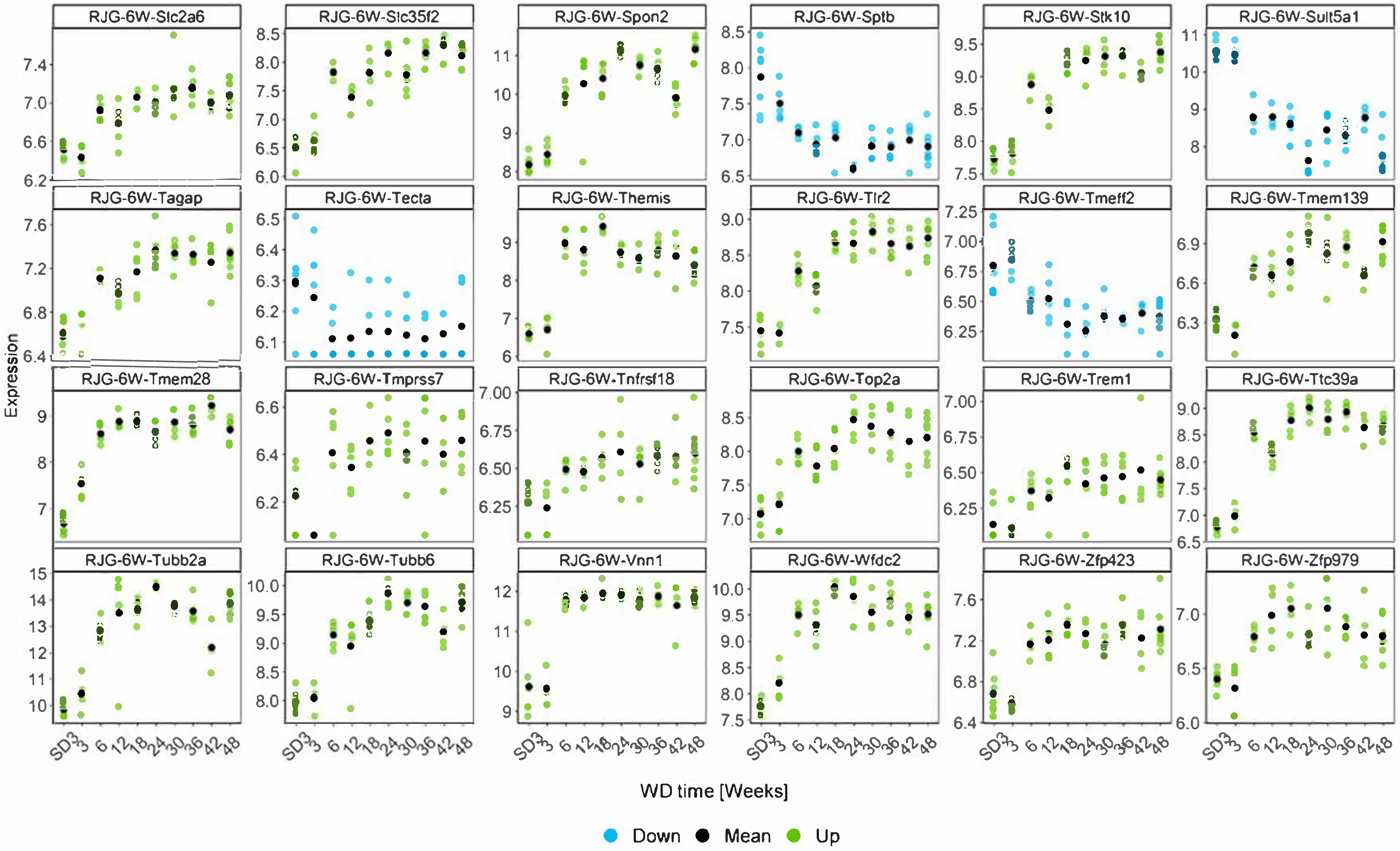

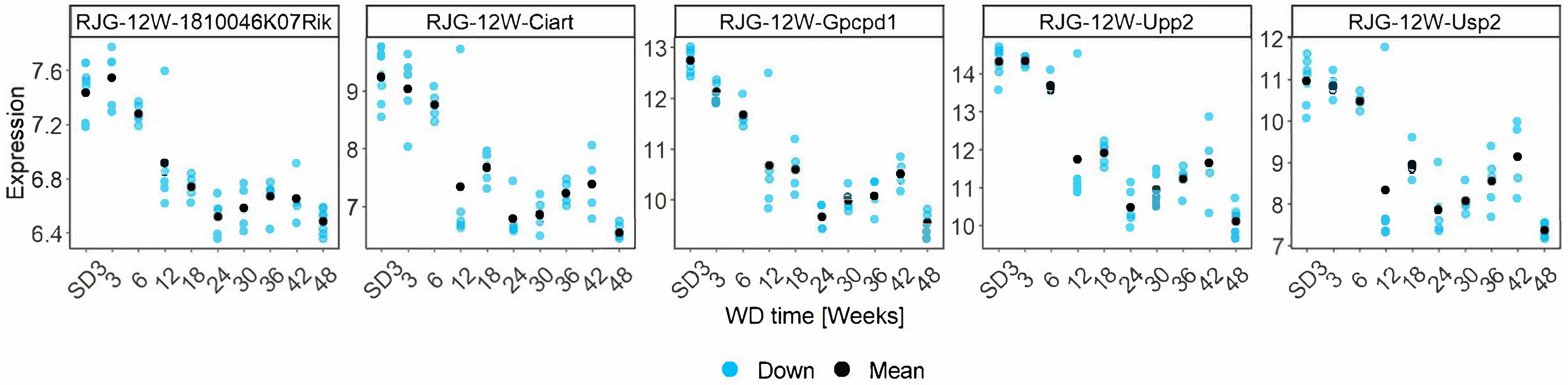

Suppl. Figure S7D. Complete set of 'rest and jump genes' (RJG) of week 12. RJG week 12 remain unaltered until week 12 of WD compared to week 3 of SD and are up- or downregulated thereafter (part 5).

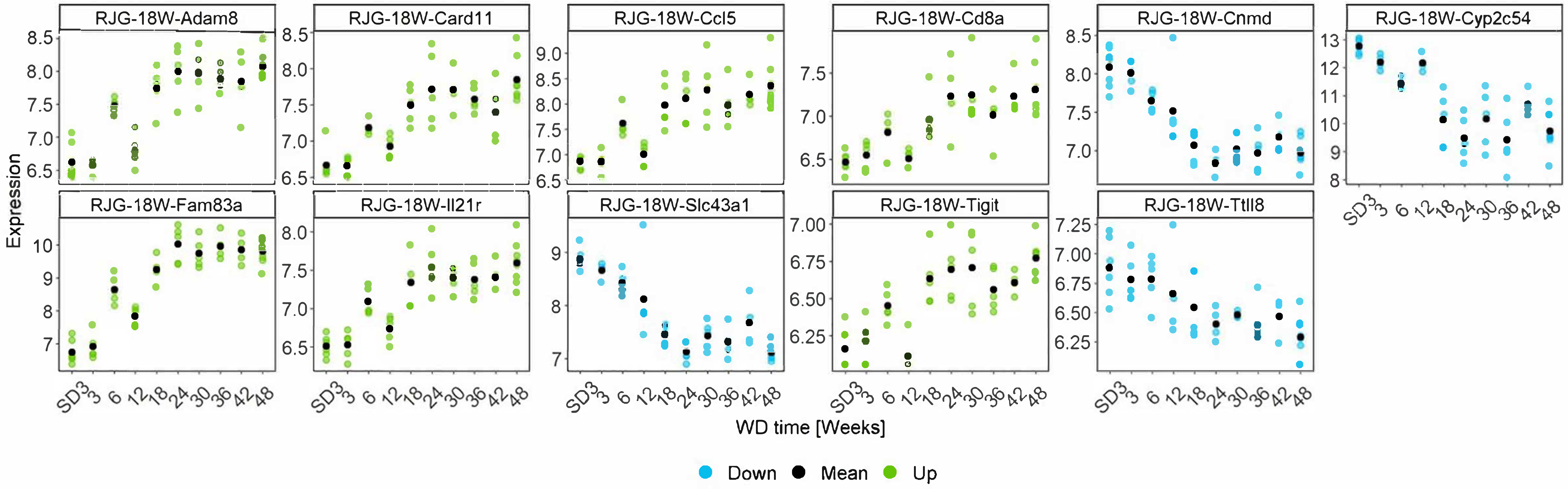

Suppl. Figure S7D. Complete set of 'rest and jump genes' (RJG) of week 18. RJG week 18 remain unaltered until week 18 of WD compared to week 3 of SD and are up- or downregulated thereafter (part 6).

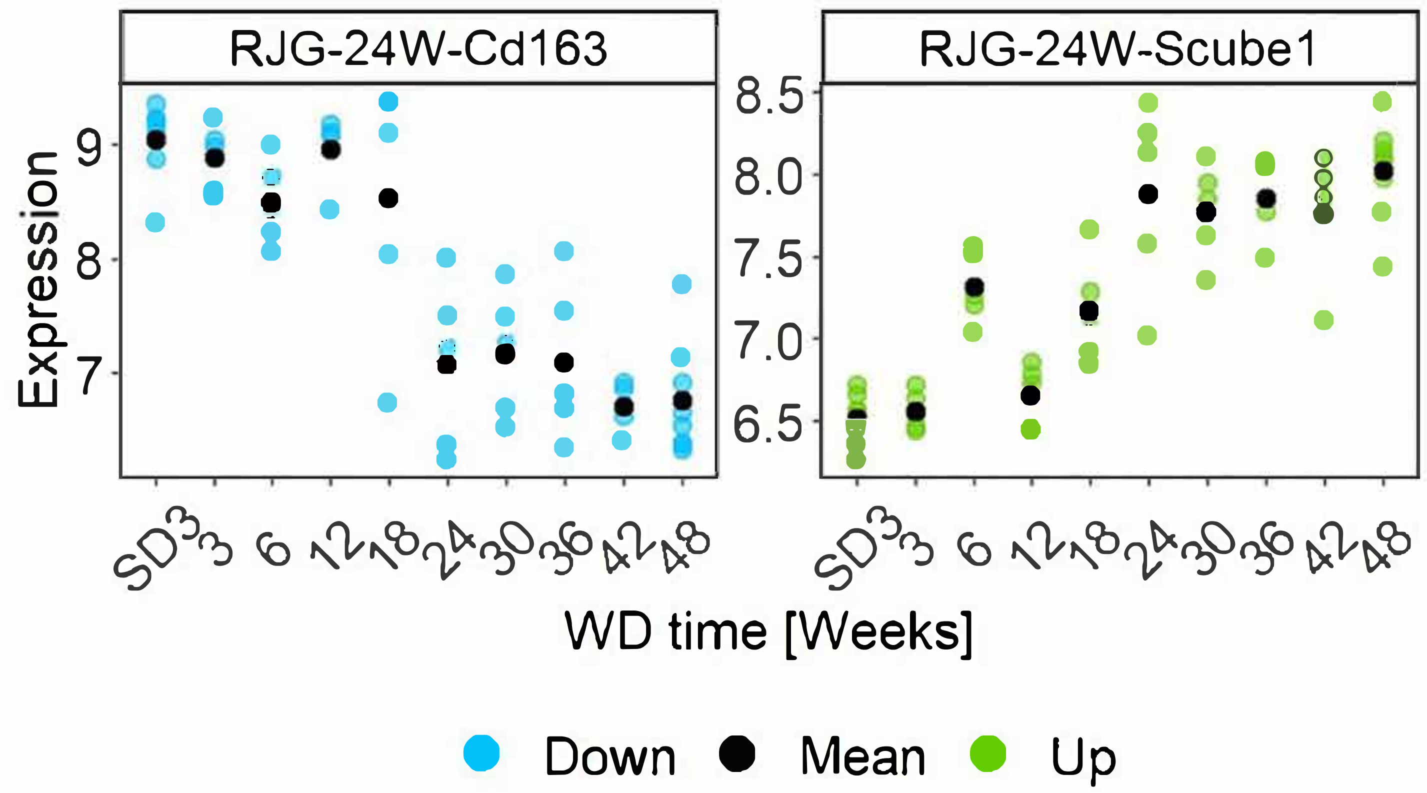

Suppl. Figure S7D. Complete set of ‘rest and jump genes‘ (RJG) of week 24. RJG week 24 remain unaltered until week 24 of WD compared to week 3 of SD and are up- or downregulated thereafter (part 7). No RJG were identified for mice fed a WD for 30 to 48 weeks.

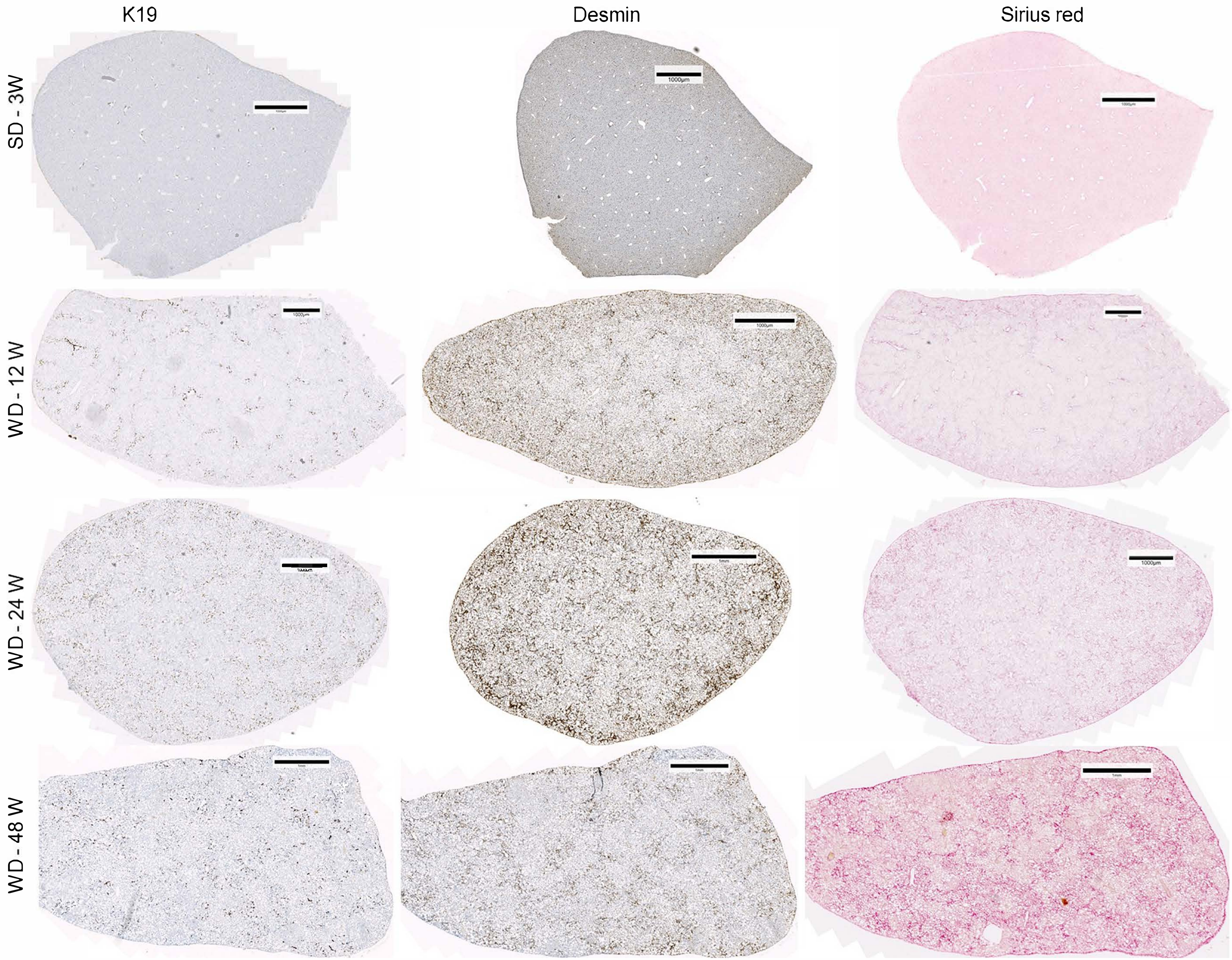

Suppl. Figure S8. Whole slide scans from the livers of standard diet- (SD) fed mice for 3 weeks and at different time intervals after western diet (WD) feeding showing the progression of ductular reaction (CK19 staining) and fibrosis (desmin and Sirius red staining).

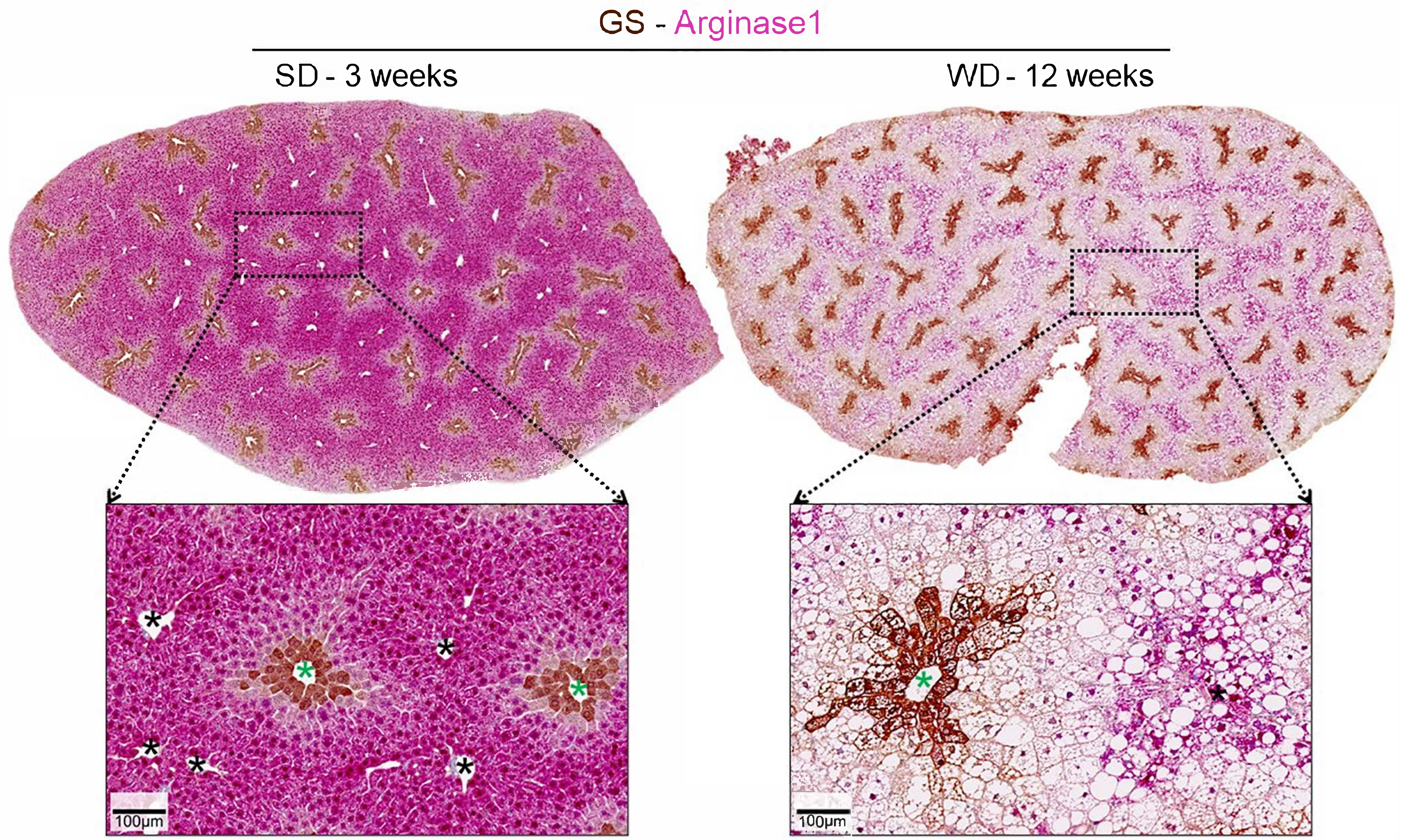

Suppl. Figure S9. Co-staining of glutamine synthetase (GS) and arginase1 in the livers of standard (SD) and western diet (WD) fed mice. The liver of the SD-fed mouse shows complementary expression of GS and arginase1; in contrast, a GS and arginase1 negative zone can be seen in the liver of the WD-fed mouse. Green asterisks indicate central veins; black asterisks indicate portal veins.

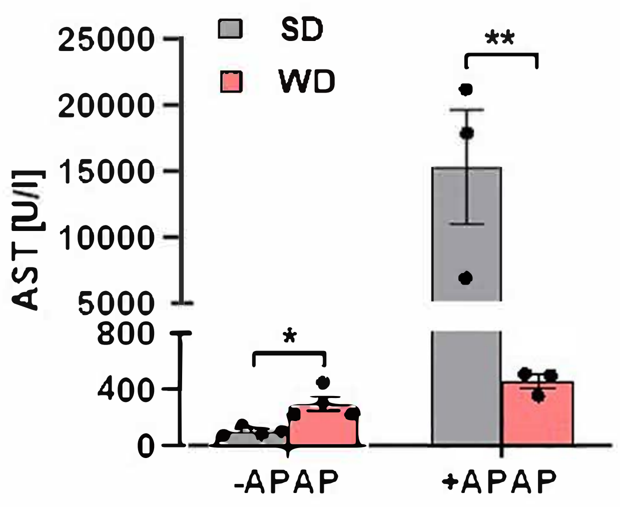

Suppl. Figure S10. Hepatotoxicity of 300 mg/kg APAP in mice fed a SD or a WD for ~50 weeks as evidenced by aspartate transaminase (AST) activity in
heart blood. Data represent mean and standard error of 3-4 mice per group. *: p<0.05; **: p<0.01 compared to SD, Tukey's multiple comparisons test; data of individual mice are illustrated by dots.

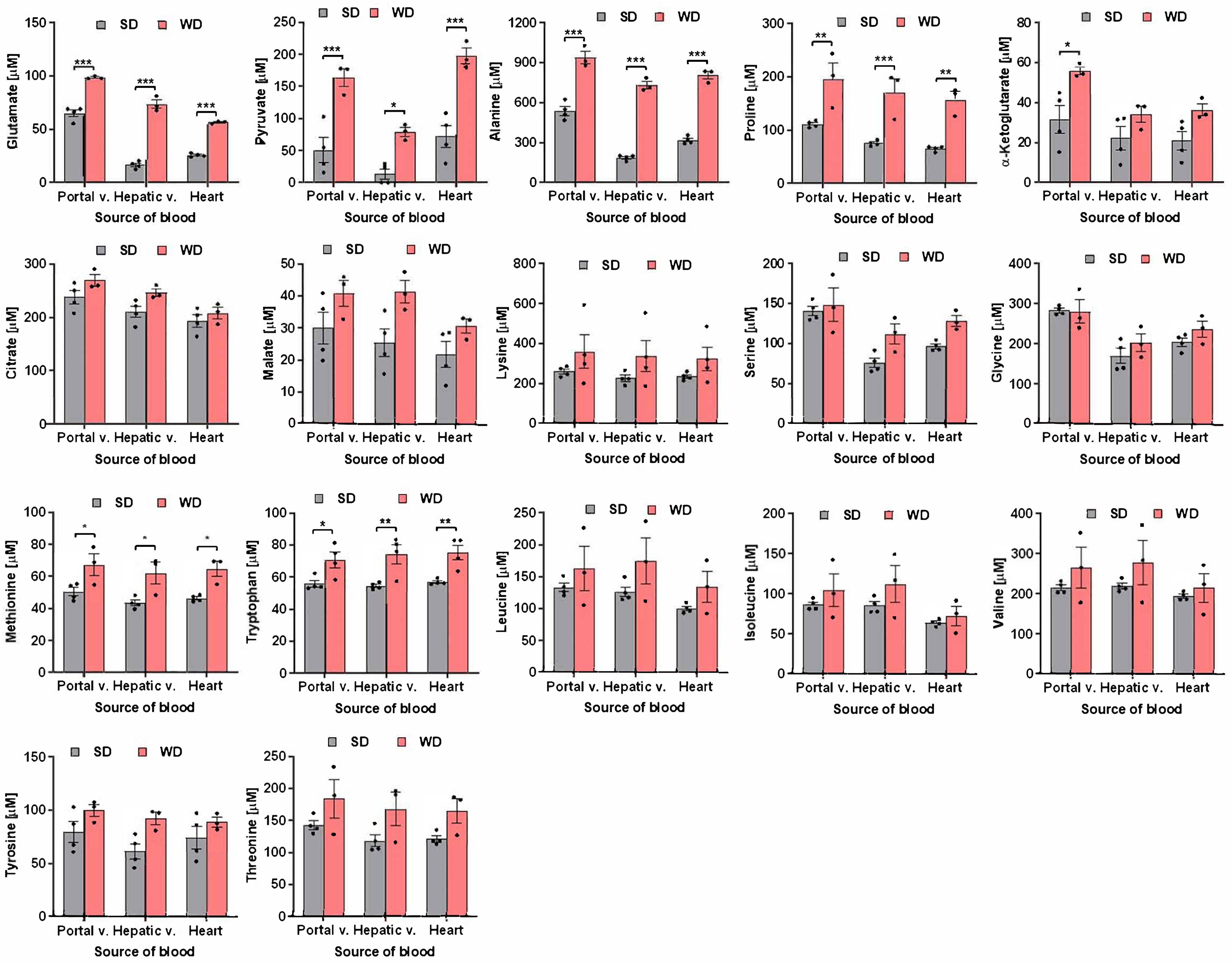

Suppl. Figure S11. Functional consequences of WD feeding (42 weeks) on amino acids and citric acid cycle intermediates as well as metabolites. Data represent mean and standard error of 4 mice per group. *: p<0.05; **: p<0.01; ***: p<0.001 compared to SD, Sidak's multiple comparisons test; data of individual mice are illustrated by dots. SD: standard diet; WD: western diet;

Supplemental video legends

Suppl. Videos S1 and S2. Intravital visualization of lipid droplets using the lipid dye bodipy (green) at 9 (Suppl. Video 1) and 30 (Suppl. Video 2) weeks after western diet (WD) feeding. Differentiation of the periportal and the pericentral lobular zones was achieved using the mitochondrial dye TMRE that leads to a stronger signal in the periportal than the pericentral zone.

Suppl. Videos S3 and S4A. Intravital imaging of livers of WD-fed mice after intravenous injection of a fluorophore-coupled F4/80 antibody (red), the mitochondrial membrane potential marker Rhodamine123 and Hoechst for nuclear staining. Suppl. Video 3. shows Kupffer cells (red) in the sinusoidal wall of a mouse fed on WD for 3 weeks. Suppl. Video 4A. A WD-fed mouse for 32 weeks showing a vital steatotic hepatocyte with mitochondrial and nuclear structures surrounded by F4/80 positive macrophages (white circle), and a lipid droplet enclosed by macrophages without discernible mitochondria or nuclear signal (pink circle).

Suppl. Video S4B. Intravital imaging of the liver of a WD-fed mouse for 24 weeks after intravenous injection of the mitochondrial membrane potential marker TMRE (red), the lipid dye bodipy (green) and the nuclear dye Hoechst (blue), showing a lipid droplet enclosed by macrophages without discernible mitochondria or nuclear signal (circle).
